# A Novel Role for NUAK1 in Promoting Ovarian Cancer Metastasis through Regulation of Fibronectin Production in Spheroids

**DOI:** 10.3390/cancers12051250

**Published:** 2020-05-15

**Authors:** Jamie Lee Fritz, Olga Collins, Parima Saxena, Adrian Buensuceso, Yudith Ramos Valdes, Kyle E. Francis, Kevin R. Brown, Brett Larsen, Karen Colwill, Anne-Claude Gingras, Robert Rottapel, Trevor G. Shepherd

**Affiliations:** 1 The Mary & John Knight Translational Ovarian Cancer Research Unit, London Regional Cancer Program, London, ON N6A 4L6, Canada; jfritz5@uwo.ca (J.L.F.); olga.collins@gmail.com (O.C.); psaxena2@uwo.ca (P.S.); adrianvincent@gmail.com (A.B.); yudithramos@yahoo.es (Y.R.V.); 2 Department of Anatomy & Cell Biology, Schulich School of Medicine and Dentistry, Western University, London, ON N6A 3K7, Canada; 3 Princess Margaret Cancer Centre, Toronto, ON M5G 2C1, Canada; kyle.francis@uhnresearch.ca (K.E.F.); rottapel@gmail.com (R.R.); 4 Terrence Donnelly Centre for Cellular and Biomolecular Research, University of Toronto, Toronto, ON M5S 3E1, Canada; bioboy99@gmail.com; 5 Lunenfeld-Tanenbaum Research Institute, Sinai Health System, Toronto, ON M5G 1X5, Canada; larsen@lunenfeld.ca (B.L.); colwill@lunenfeld.ca (K.C.); gingras@lunenfeld.ca (A.-C.G.); 6 Department of Molecular Genetics, University of Toronto, Toronto, ON M5S 1A8, Canada; 7 Department of Obstetrics & Gynaecology, Schulich School of Medicine and Dentistry, Western University, London, ON N6A 4L6, Canada; 8 Department of Oncology, Schulich School of Medicine & Dentistry, The University of Western Ontario, London, ON N6A 4L6, Canada

**Keywords:** ovarian cancer, spheroid, LKB1, NUAK1, metastasis, fibronectin

## Abstract

Epithelial ovarian cancer (EOC) has a unique mode of metastasis, where cells shed from the primary tumour, form aggregates called spheroids to evade anoikis, spread through the peritoneal cavity, and adhere to secondary sites. We previously showed that the master kinase Liver kinase B1 (LKB1) is required for EOC spheroid viability and metastasis. We have identified novel (nua) kinase 1 (NUAK1) as a top candidate LKB1 substrate in EOC cells and spheroids using a multiplex inhibitor beads-mass spectrometry approach. We confirmed that LKB1 maintains NUAK1 phosphorylation and promotes its stabilization. We next investigated NUAK1 function in EOC cells. Ectopic NUAK1-overexpressing EOC cell lines had increased adhesion, whereas the reverse was seen in OVCAR8-*NUAK1*KO cells. In fact, cells with NUAK1 loss generate spheroids with reduced integrity, leading to increased cell death after long-term culture. Following transcriptome analysis, we identified reduced enrichment for cell interaction gene expression pathways in OVCAR8-*NUAK1*KO spheroids. In fact, the *FN1* gene, encoding fibronectin, exhibited a 745-fold decreased expression in *NUAK1*KO spheroids. Fibronectin expression was induced during native spheroid formation, yet this was completely lost in *NUAK1*KO spheroids. Co-incubation with soluble fibronectin restored the compact spheroid phenotype to OVCAR8-*NUAK1*KO cells. In a xenograft model of intraperitoneal metastasis, NUAK1 loss extended survival and reduced fibronectin expression in tumours. Thus, we have identified a new mechanism controlling EOC metastasis, through which LKB1-NUAK1 activity promotes spheroid formation and secondary tumours via fibronectin production.

## 1. Introduction

Epithelial ovarian cancer (EOC) is the most lethal gynecologic malignancy in the developed world and it is characterized by early and rapid metastasis [1]. Most women are diagnosed with advanced-stage disease with a five-year survival rate of only 29% [2]. The standard treatment plan for patients with late-stage EOC is maximal surgical cytoreduction with adjuvant chemotherapy of carboplatin and paclitaxel [1]. However, the majority of patients will eventually develop disease recurrence and resistance to chemotherapy. Therefore, there is a need to develop novel therapeutic strategies in order to impede EOC metastasis [3].

In EOC metastasis, malignant cells shed from the primary ovarian tumour and spread into the peritoneal cavity [4]. Ascites commonly accumulates in the peritoneal cavity of patients with advanced-stage disease. In the ascites fluid, cancer cells form multi-cellular structures that are known as spheroids to evade anoikis, a form of apoptosis due to loss of cell attachment. Eventually, spheroids will adhere to the peritoneum to continue to invade at secondary sites [5,6]. In addition to playing a key role in efficient peritoneal metastasis, spheroids acquire resistance to chemotherapy due to the acquisition of cellular quiescence [7,8].

The formation of spheroids is controlled by several cell attachments through cadherins and indirectly through extracellular matrix (ECM) proteins and integrins [9,10,11]. Fibronectin exists as a soluble form in the plasma and as an insoluble fibrillar form in the ECM [12]. Fibronectin is a ligand for multiple integrins; however, its canonical receptor is the α5β1integrin heterodimer. In EOC, the interaction between fibronectin and α5β1 integrins is necessary for efficient spheroid formation [11]. In fact, elevated fibronectin expression is correlated with a worsened tumour stage and decreased overall survival in EOC patients [13]. In mouse models, fibronectin loss reduces EOC cell adhesion, invasion, and metastatic potential [14].

The elucidation of key intracellular signaling pathways in ovarian cancer spheroids would allow for an improved understanding of metastatic processes and would aid in the identification of novel therapeutic targets. Our group previously showed that Liver kinase B1 (LKB1) is critical for ovarian cancer metastasis [15,16]. LKB1, which is encoded by *STK11*, is a serine-threonine kinase that is known best as having tumour suppressive-like activity in cancers [17]. *STK11* inactivating mutations lead to Peutz–Jeghers syndrome, a condition that is characterized by gastrointestinal polyps and increased risk for cancer [18]. However, we have shown that LKB1 activity is intact and facilitates tumour progression in late-stage EOC [15,16]. LKB1 is expressed in established EOC cell lines, patient-derived ascites cells, and tumour extracts [16]. In addition, sustained LKB1 loss decreases the anchorage-independent growth of EOC cells and decreases spheroid integrity and cell viability [15]. LKB1 loss extends survival and decreases tumour burden in a xenograft model of intraperitoneal metastasis [15].

The canonical downstream target of LKB1 is AMP-activated protein kinase (AMPK), a regulator of metabolic stress [17]. Interestingly, our group showed that LKB1’s pro-metastatic role in EOC occurs independent of AMPK activity [15]. LKB1 is known as a master upstream kinase by its regulation of 12 other AMPK-related kinases (ARKs): brain-specific kinases 1 and 2 (BRSK1/2), novel (nua) kinases 1 and 1 (NUAK1/2), salt-inducible kinases 1, 2, and 3 (SIK1/2/3), microtubule-affinity regulating kinases 1, 2, 3, and 4 (MARK1/2/3/4), and SNF-related serine/threonine-protein kinase (SNRK) [19]. Herein, we used a multiplex inhibitor bead-mass spectrometry analysis in order to identify NUAK1 as the most likely ARK family member substrate enabling LKB1 to drive EOC metastasis.

NUAK1 is a serine-threonine kinase that can be phosphorylated by LKB1 at a conserved threonine 211 residue on the T-loop of its catalytic domain [19,20]. Prior studies have shown that NUAK1 has pro-tumorigenic functions. NUAK1 promotes cancer cell survival by inhibiting apoptosis and inducing the S-phase in the cell cycle. It can also protect tumours from oxidative stress by increasing nuclear translocation of the anti-oxidant regulator, Nrf2 [21]. Previous studies also suggest that NUAK1 impacts cell adhesion by increasing epithelial–mesenchymal transition (EMT) and stimulating cell detachment via myosin phosphatase complex regulation [22,23]. A tumour-promoting role for NUAK1 is strengthened by studies where elevated NUAK1 correlates with poor prognosis in several malignancies, including EOC [21,24].

In this study, we aimed to further elucidate the role of the LKB1 target NUAK1 in EOC metastasis. We show that LKB1 regulates NUAK1 expression, phosphorylation, and stability in EOC cells and spheroids. NUAK1 controls key steps of the metastatic cascade by regulating EOC cell adhesion and spheroid integrity via fibronectin expression and resultant deposition in order to promote spheroid formation. Furthermore, NUAK1 loss in a xenograft model of intraperitoneal metastasis extended host survival and reduced fibronectin expression in tumours.

## 2. Results

### 2.1. NUAK1 Expression is Regulated by LKB1 in EOC

**Figure 1:**
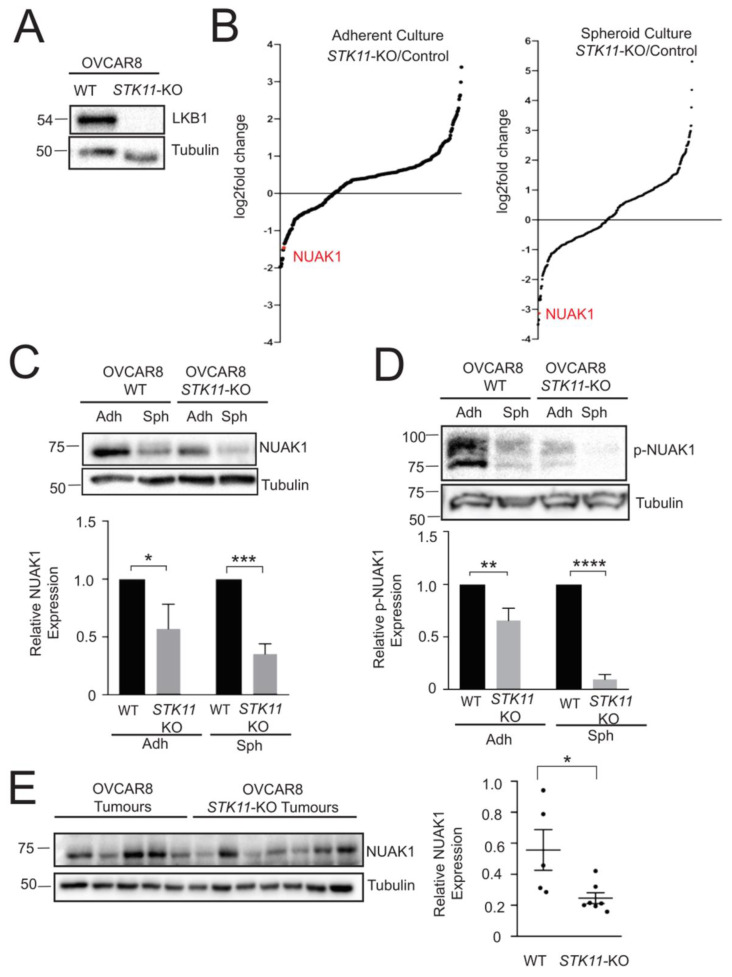
NUAK1 expression is regulated by LKB1 in epithelial ovarian cancer (EOC) spheroids and xenograft tumours. (**A**) Western blot analysis of OVCAR8 parental and OVCAR8-*STK11*KO cells to confirm LKB1 loss by CRISPR/Cas9 genome editing. Whole blot images can be found in Figures S1 and S2. (**B**) Multiplexed kinase inhibitor bead-mass spectrometry analysis was completed using OVCAR8-*STK11*KO and OVCAR8 cells. Log_2_-fold change of differentially expressed kinases is presented for OVCAR8-*STK11*KO versus OVCAR8 cells for adherent and spheroid cultures. (**C**) Immunoblot analysis to determine NUAK1 levels in OVCAR8 and OVCAR8-*STK11*KO cells cultured as adherent cells (Adh) or spheroids (Sph) for 72 h. Tubulin was used as a loading control. Densitometric analysis of NUAK1 expression relative to tubulin and normalized to OVCAR8 adherent cells and multiple *t*-test with Bonferroni correction was performed (* *p* < 0.05; *** *p* < 0.001; n = 3). Whole blot images can be found in Figures S3 and S4. (**D**) Immunoblot analysis was completed using Phostag^TM^ acrylamide gels to determine phosphorylated NUAK1 levels in OVCAR8 and OVCAR8-*STK11*KO cells cultured as adherent cells (Adh) and spheroids (Sph) for 72 h. Tubulin was used as a loading control. Densitometric analysis of phospho-NUAK1 expression relative to tubulin and normalized to OVCAR8 cells and multiple *t*-test with Bonferroni correction was performed (** *p* < 0.01; **** *p* < 0.0001; n = 3). Whole blot images can be found in Figures S5 and S6. (**E**) Immunoblot analysis of NUAK1 expression in OVCAR8 and OVCAR8-*STK11*KO xenograft tumours. Densitometric analysis of NUAK1 expression relative to tubulin for OVCAR8 tumours (n = 5) and OVCAR8-*STK11*KO tumours (n = 7). Statistical analysis was performed using two-tailed Student’s *t*-test (* *p* < 0.05). Whole blot images can be found in Figures S7 and S8.

We performed multiplex inhibitor beads-mass spectrometry (MIB/MS) to elucidate alternative LKB1 substrates in EOC since we previously demonstrated that LKB1 is required for efficient EOC metastasis, yet acts independently from its canonical target AMPK [15,16]. Briefly, several broad-acting ATP-competitive kinase inhibitors are immobilized to beads to capture active kinases present in protein lysates, which is then coupled with tandem mass spectrometry to identify and quantify eluted kinases [25]. Our MIB/MS analysis was completed using OVCAR8 and OVCAR8-*STK11*KO cells that were previously generated using CRISPR/Cas9 editing [15] (Figure 1A). Out of 12 ARKs, NUAK1 was the only family member that was significantly decreased in *STK11*KO adherent cells and spheroids (Figure 1B). In fact, it was the second most down-regulated kinase in spheroids (−8.75-fold change) and third most-down-regulated kinase (−2.8-fold change) in adherent cells (Tables S2 and S3).

We assessed NUAK1 expression by immunoblot analysis and observed a significant decrease in NUAK1 expression levels in OVCAR8-*STK11*KO spheroids to confirm our *MIB/MS* results (Figure 1C). NUAK1 phosphorylation was examined to further study the regulation of NUAK1 by LKB1. NUAK1 is directly phosphorylated at Ser211 by LKB1 [17,20]; however, there are no commercially available antibodies for this modification. Thus, we employed Phostag^TM^ acrylamide gels [26] and observed a significant decrease in phospho-NUAK1 due to LKB1 loss in OVCAR8 cells in both adherent and spheroid culture conditions (Figure 1D). Thus, NUAK1 expression and phosphorylation require LKB1 in EOC cells and spheroids.

Finally, we sought whether LKB1 regulates NUAK1 expression in tumours. While using xenograft tumour samples collected from our previous study [15], there was a significant decrease in NUAK1 protein expression in OVCAR8-*STK11*KO tumours as compared with OVCAR8 tumours (Figure 1E). Altogether, our findings suggest that LKB1 regulates NUAK1 expression in ovarian cancer spheroids and tumour samples.

### 2.2. NUAK1 Is Differentially Expressed during Spheroid Formation

**Figure 2:**
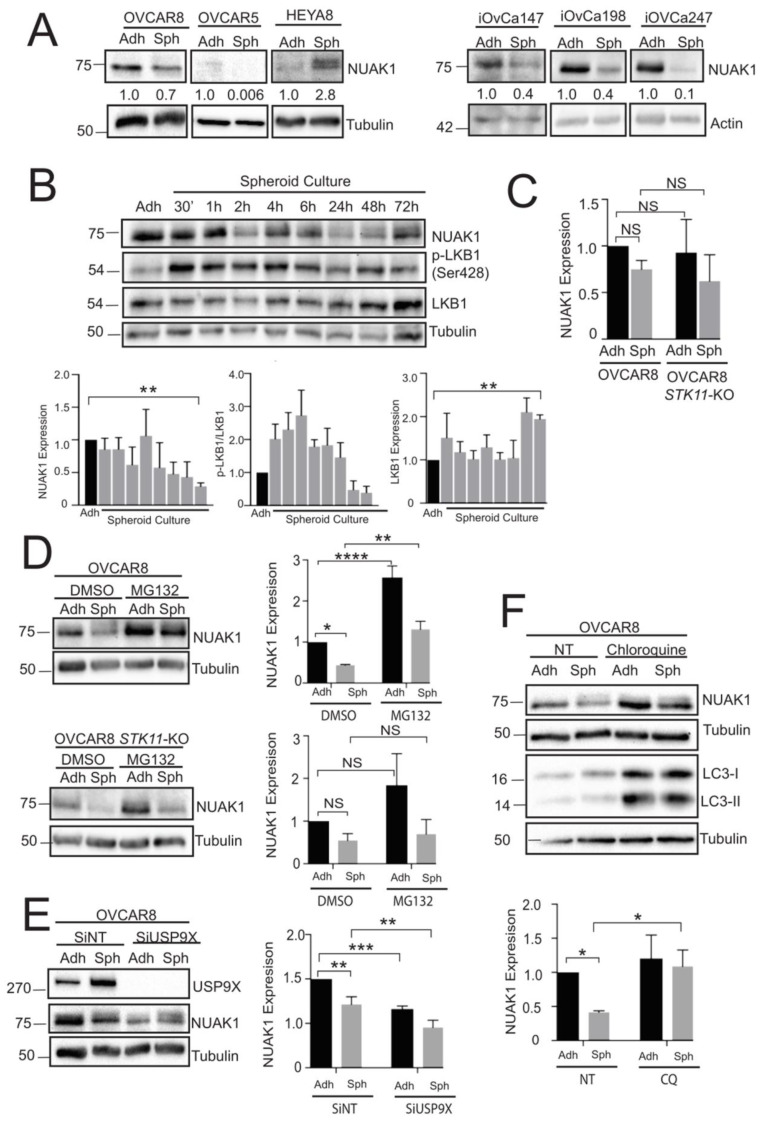
Regulation of NUAK1 expression and stability in EOC spheroids. (**A**) Immunoblot analysis to assess NUAK1 expression in HGSOC cell lines (OVCAR8, OVCAR5), a non-HGSOC cell line (HEYA8), and patient-derived ascites cell lines (iOvCa147, iOvCa198, iOvCa247) cultured under adherent (Adh) or suspension (Sph) conditions. Tubulin and actin were used as loading controls. Fold change in NUAK1 expression relative to adherent cells is indicated. Whole blot images can be found in Figures S9–S18. (**B**) Time course analysis of NUAK, phospho-LKB1 (S428), and total LKB1 during OVCAR8 spheroid formation. Densitometric analysis for NUAK1 relative to tubulin, phospho-LKB1 relative to LKB1, and LKB1 relative to tubulin. One-way ANOVA and Dunnett’s multiple comparison test were performed (** *p* < 0.01; n = 3). Whole blot images can be found in Figures S19–S22. (**C**) RT-qPCR analysis of *NUAK1* gene expression in OVCAR8 and OVCAR8-*STK11*KO cells cultured under adherent conditions (Adh) or as spheroids (Sph). Gene expression is relative to *GADPH* and normalized to OVCAR8 adherent cells. Two-way ANOVA and Tukey’s multiple comparisons test was performed (NS = non-significant; n = 3). (**D**) Immunoblot analysis of NUAK1 expression in OVCAR8 and OVCAR8-*STK11*KO cells treated with 10 μM MG132 for 8 h, or 0.1% DMSO as a control. The cells were cultured in adherent conditions (Adh) or as spheroids (Sph). Densitometric analysis of NUAK1 relative to tubulin and normalized to DMSO-treated adherent cells. Two-way ANOVA and Tukey’s multiple comparisons test were performed (NS = not significant; * *p* < 0.05; ** *p* < 0.01; **** *p* < 0.0001; n = 3). Whole blot images can be found in Figures S23 and S24. (**E**) Immunoblot analysis of NUAK1 and USP9X expression in OVCAR8 cells transfected with control siRNA (siNT) or siRNA targeting *USP9X*. Cells were cultured in adherent conditions (Adh) or as spheroids (Sph). Densitometric analysis of NUAK1 relative to tubulin and normalized to siNT-transfected controls. Two-way ANOVA and Tukey’s multiple comparisons test was performed (** *p* < 0.01; *** *p* < 0.001; n = 3). Whole blot images can be found in Figures S25–S27. (**F**) Immunoblot analysis of NUAK1 and LC3-I/II expression in OVCAR8 cells treated with 25 μM chloroquine for 8 h or left untreated. Cells were cultured in adherent conditions (Adh) or as spheroids (Sph). Densitometric analysis of NUAK1 relative to tubulin and normalized to untreated controls. Two-way ANOVA and Tukey’s multiple comparisons test was performed (* *p* < 0.05; n = 3). Whole blot images can be found in Figures S28–S31.

Using a spheroid model of metastasis, we sought to determine whether NUAK1 expression changes during spheroid formation. To achieve this, we commonly compare protein expression between proliferative adherent cells and quiescent spheroids [7]. By examining spheroids generated from high-grade serous ovarian cancer (HGSOC) cells lines OVCAR8 and OVCAR5 [27,28], we found that NUAK1 protein levels are down-regulated in spheroids when compared to adherent cells (Figure 2A). This trend was also apparent in multiple patient ascites-derived cell lines cultured as spheroids, including cells from a Stage IIIB HGSOC patient who had received six cycles of carboplatin and paclitaxel (iOvCa198) and upon platinum resistance (iOvCa247; Figure 2A). For comparison, the non-HGSOC cell line HEYA8 [27] was examined. In contrast to HGSOC spheroids, HEYA8 spheroids showed increased NUAK1 as compared to adherent cells (Figure 2A).

Time course analysis was completed in OVCAR8 spheroids to study these changes in NUAK1 expression in greater detail. During early spheroid formation, NUAK1 expression is relatively high and this parallels phospho-LKB1 Ser428 levels in spheroids (Figure 2B). The NUAK1 levels decrease significantly when compared to adherent cells later during spheroid formation and again this correlates with reduced phospho-LKB1 with a statistically significant decrease observed by 72 h. NUAK1 maintains a low yet detectable level at these later points.

To investigate this increase in NUAK1 in EOC spheroids, we first performed RT-qPCR to determine whether *NUAK1* is down-regulated at the transcript level. There was no significant difference in *NUAK1* gene expression between adherent cells and spheroids (Figure 2C). Furthermore, there was no significant difference in *NUAK1* mRNA between OVCAR8 and OVCAR8-*STK11*KO cells and spheroids. These results suggest that down-regulated NUAK1 expression is controlled at the protein level. Previous studies have shown that the ubiquitin-proteasome system (UPS) can regulate NUAK1 [29]. We treated cells and spheroids with the proteasome inhibitor, MG132, to test whether the UPS controls NUAK1 expression in EOC. The addition of MG132 to OVCAR8 cells not only prevented the NUAK1 decrease observed in spheroids, but it significantly increased its levels (Figure 2D). This effect of proteasome inhibition on NUAK1 was observed in both adherent cells (MG132-treated:DMSO-treated ratio of 1.98) and spheroids (ratio of 1.75). Intriguingly, MG132 did not increase NUAK1 expression in OVCAR8 spheroids lacking LKB1 (Figure 2D), in which we observed a ratio of 0.74 as compared with 1.51 for OVCAR8-*STK11*KO adherent cells. These results support the importance of LKB1 in regulating NUAK1 expression levels in spheroids, and that the UPS contributes to its down-regulation.

A previous report demonstrated that the activity of Ubiquitin Specific Peptidase 9 X-Linked (USP9X) removed NUAK1 ubiquitination [30]. Further to our result of the UPS controlling NUAK1 expression in EOC, we tested whether USP9X can regulate NUAK1 in spheroids. We predicted that *USP9X* knockdown would lead to decreased NUAK1 via proteasomal degradation. Indeed, *USP9X* knockdown significantly decreased NUAK1 in OVCAR8 adherent cells and spheroids (Figure 2E).

Spheroids were treated with the lysosomotropic autophagy inhibitor, Chloroquine (CQ), to determine whether lysosomal degradation also contributes to decreased NUAK1. CQ treatment led to a significant increase in NUAK1 in OVCAR8 spheroids, where there was a CQ-to-control ratio of 2.63 as compared with a ratio of only 1.20 for adherent cells (Figure 2F). We observed the buildup in LC3-I and -II proteins, as expected for late-stage autophagy inhibition with this agent [31]. Altogether, these findings suggest that protein stability in EOC cells and spheroids primarily controls NUAK1 protein levels.

### 2.3. NUAK1 Promotes EOC Cell Adhesion and Spheroid Integrity

**Figure 3:**
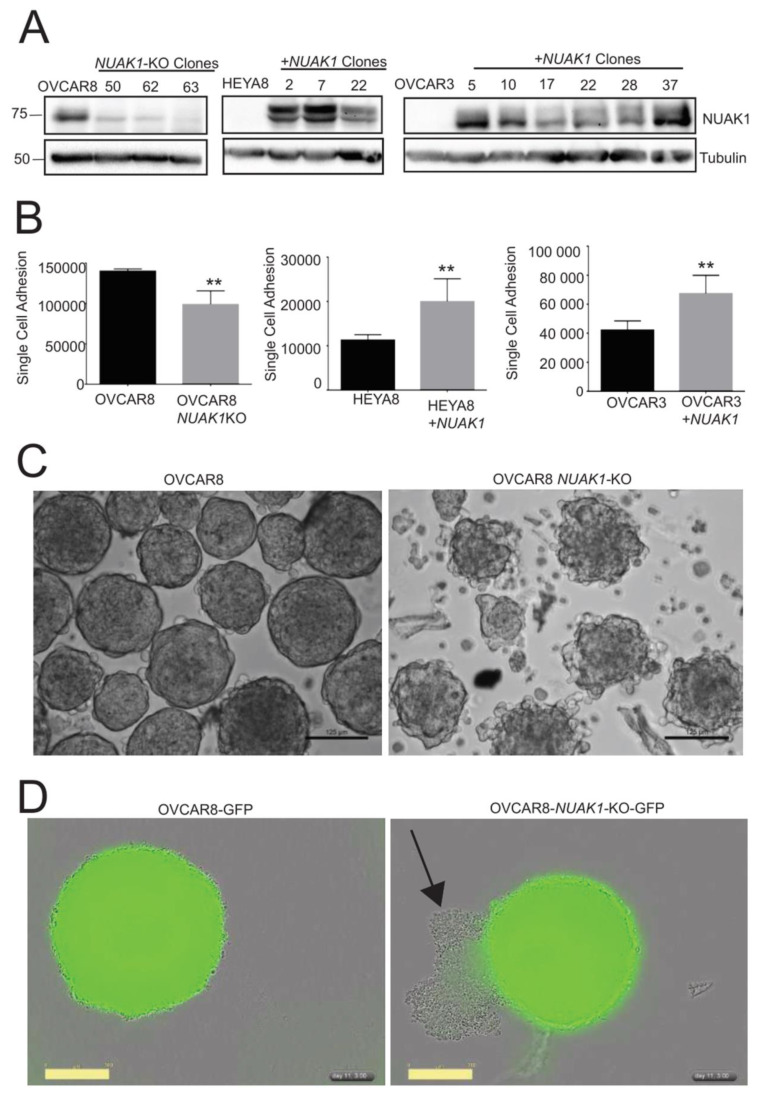
NUAK1 regulates EOC cell adhesion and spheroid integrity. (**A**) Immunoblot analysis of OVCAR8 − *NUAK1*KO cells, and HEYA8 + NUAK1 and OVCAR3 + NUAK1 overexpressing cells and matched parental cell lines. Tubulin was used as a loading control. Whole blot images can be found in Figures S32–S37. (**B**) Single cell adhesion was quantified by Trypan Blue Exclusion cell counting for OVCAR8−*NUAK1*KO, HEYA8 + NUAK1, and OVCAR3 + NUAK1 cells and parental cell line controls. Data are presented as absolute cell counts from pooled data among multiple clones. Statistical analysis was performed using two-tailed Student’s *t*-test (** *p* < 0.01; n = 3). (**C**) Images of OVCAR8 and OVCAR8-*NUAK1*KO spheroids cultured for 11 days in ULA dishes with methylcellulose. Representative images of three independent experiments are displayed. Scale bars represent 125 μm. (**D**) Images of 11-day OVCAR8 and OVCAR8-*NUAK1*KO spheroids stably-transduced with lentivirus expressing NucLight GFP. Phase contrast and green fluorescence images were captured in real-time while using the IncuCyte Zoom imaging system. Arrow indicates cells detached from spheroid and loss of green fluorescence is evident. Representative images of three independent experiments are displayed. Scale bars represent 300 µm.

Prior studies have shown that NUAK1 plays a role in cell adhesion. NUAK1 promotes epithelial-to-mesenchymal transition (EMT) in cancer and induces cell detachment by regulating the MYPT-PP1β complex [22,24,32]. Cell adhesion is critical during EOC metastasis, because cell-ECM interactions mediate the spheroid formation and subsequent re-attachment at secondary sites [10]. We used OVCAR8, OVCAR3, and HEYA8 to further investigate this function of NUAK1 in EOC metastasis, because they readily form spheroids in vitro and can establish xenografted tumours when injected intraperitoneally into immune-compromised mice [28,33]. OVCAR8 cells express high levels of NUAK1, while OVCAR3 and HEYA8 cells express very low to undetectable NUAK1 protein (Figure 3A). We generated three independent stable clonal lines lacking intact NUAK1 expression (OVCAR8−*NUAK1*KO) while using CRISPR-Cas9 genome editing; and in a reciprocal fashion, we engineered multiple clones of OVCAR3 and HEYA8 cell lines to stably overexpress NUAK1 (OVCAR3 + NUAK1 and HEYA8 + NUAK1; Figure 3A). We performed timed adhesion assays to examine whether NUAK1 controls cell attachment. HEYA8+NUAK1 cells had significantly greater single cell adhesion when compared to HEYA8 empty-vector control cells; likewise, this was observed in OVCAR3+NUAK1 cells (Figure 3B). In contrast, OVCAR8−*NUAK1*KO cells had significantly lower cell adhesion when compared to parental OVCAR8 cells. Thus, our results support previous studies that NUAK1 enhances EOC cell adhesion.

Given this result, we next investigated whether NUAK1 is required for proper EOC spheroid formation. The cells were cultured in Ultra-Low Attachment (ULA) culture plates and methylcellulose was added to the media to facilitate spheroid formation [34]. After extensive growth in suspension culture, OVCAR8 cells formed dense spheroids as expected (Figure 3C). However, OVCAR8-*NUAK1*KO spheroids were markedly less compact and exhibited a disaggregated appearance. To further examine this altered phenotype and visualize cell viability, OVCAR8 and OVCAR8-*NUAK1*KO cells were stably transduced with NucLight GFP lentivirus. Phase contrast and green fluorescence images were captured during single spheroid culture using an IncuCyte Zoom. Using the same time point as in Figure 3C, we observed intact spheroids with robust green fluorescence signal for OVCAR8-GFP cells (Figure 3D). In contrast, OVCAR8-*NUAK1*KO-GFP spheroids showed decreased integrity with many extruding non-viable cells, as visualized by the loss of green fluorescence signal. Taken together, NUAK1 loss reduces EOC cell adhesion, leading to decreased spheroid integrity.

### 2.4. NUAK1 Promotes EOC Spheroid Formation through Fibronectin Expression

**Figure 4:**
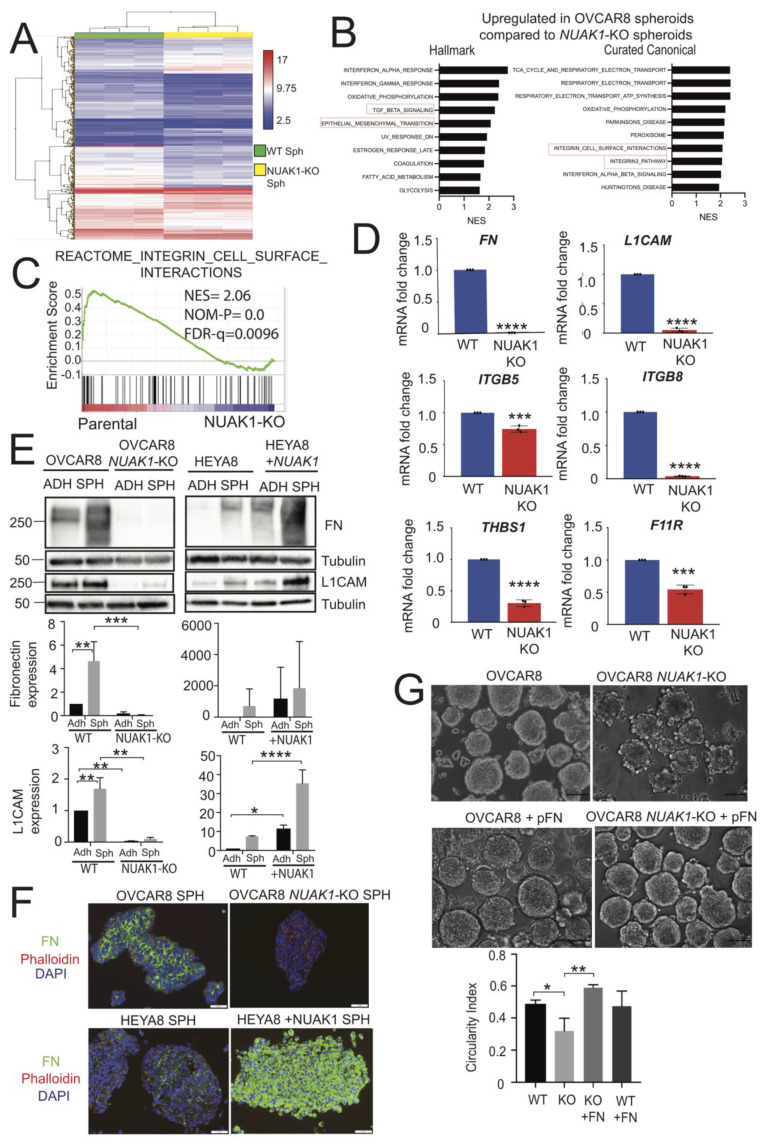
NUAK1 promotes fibronectin expression in EOC spheroids. (**A**) Hierarchical clustering heat map showing gene expression profiles for OVCAR8 and OVCAR8-*NUAK1*KO spheroids (n = 3). Up-regulated (red) and down-regulated (blue) genes with a fold change ≥ 2 or ≤ −2 and p < 0.05 are shown. (**B**) Top 10 gene sets up-regulated in OVCAR8 spheroids when compared with OVCAR8-*NUAK1*KO spheroids presented as normalized enrichment score (NES) using the GSEA Hallmark and Curated Canonical databases. (**C**) Reactome integrin cell surface interactions enrichment plot with normalized enrichment score (NES), nominal p-value, and FDR q-value are shown. (**D**) RT-qPCR validation of genes selected from the integrin cell surface interactions signature. Fold-change in mRNA levels is presented for OVCAR8 and OVCAR8-*NUAK1*KO spheroids. Statistical analysis was performed using two-tailed Student’s *t*-test (*** *p* < 0.001; **** *p* < 0.0001; n = 3). (**E**) Immunoblot analysis of fibronectin and L1CAM in OVCAR8 − *NUAK1*KO and HEYA8 + NUAK1 cells with respective parental cell lines cultured in adherent conditions (ADH) or in suspension (SPH). Tubulin was used as a loading control. Densitometric analysis of fibronectin and L1CAM relative to tubulin, normalized to adherent cells. Two-way ANOVA and Tukey’s multiple comparisons test was performed (* *p* < 0.05; ** *p* < 0.01; *** *p* < 0.001; **** *p* < 0.0001; n = 3). Whole blot images can be found in Figures S38–S45. (**F**) Immunofluorescence analysis of fibronectin (green) in OVCAR8 − *NUAK1*KO and HEYA8 + NUAK1 spheroids with respective parental cell lines. Phalloidin (red) and DAPI (blue) were used as actin cytoskeleton and nuclear stains, respectively. Images were captured using Olympus AX70 upright microscope and ImagePro image capture software; representative images from three independent experiments are shown. Scale bar represents 100 μm. (**G**) Images of OVCAR8 and OVCAR8-*NUAK1*KO spheroids cultured for 11 days in ULA dishes with methylcellulose and supplemented with or without 5 µg/mL plasma fibronectin (pFN) captured using a Leica inverted light microscope. Representative images from three independent experiments are shown. Scale bars represent 100 μm. Circularity index was measured and calculated using Fiji as described in Materials & Methods. One-way ANOVA and Tukey’s multiple comparisons test was performed (* *p* < 0.05; ** *p* < 0.01; n = 3).

We opted to perform global transcriptome analysis to understand the molecular basis for the impaired spheroid integrity in OVCAR8-*NUAK1*KO spheroids. A total of 606 genes were differentially expressed (fold change ≥ 2 or ≤ −2) at 24 h between OVCAR8 and OVCAR8-*NUAK1*KO spheroids while using the Affymetrix Human Clariom S microarray (Table S4). Hierarchical clustering demonstrated that there were distinct gene expression profiles between OVCAR8 and OVCAR8-*NUAK1*KO spheroids (Figure 4A). Gene Set Enrichment analysis (GSEA) using this transcriptome data was completed to determine potential mechanisms controlled by NUAK1. Using the Hallmark and Curated Canonical databases in GSEA, it was revealed that the interferon, metabolism, and EMT signatures were enriched in OVCAR8 spheroids when compared with OVCAR8-*NUAK1*KO (Figure 4B; Tables S5 and S6), which supports a previous study showing NUAK1 can promote EMT in EOC cells [23]. Interestingly, we found multiple pathways involving integrin-mediated cell attachment that were decreased due to NUAK1 loss in spheroids (Figure 4C; Table S7), thus supporting our in vitro cell adhesion results.

Individual genes with a greater than two-fold change from this dataset were chosen to validate by RT-qPCR. While *L1CAM* was not a core enriched gene within the Reactome Integrin Cell Surface Interaction dataset, it was included, since this transmembrane adhesion molecule promotes EOC spheroid formation in concert with fibronectin [35]. Furthermore, it was the 10th most down-regulated gene in OVCAR8−*NUAK1*KO spheroids out of all differentially expressed genes (Table S4). We observed a significant decrease in *FN1* mRNA levels in OVCAR8-*NUAK1*KO spheroids, and a coordinate reduction in *L1CAM* mRNA (Figure 4D). Interestingly, while there was no change in expression level of the canonical fibronectin receptor genes, *ITGA5* and *ITGB1*, there was a significant decrease in other integrins, namely *ITGB5* and *ITGB8*, as well as the adhesion molecules thrombospondin 1 (*THBS1*) and F11 receptor (*F11R*).

We first assessed fibronectin and L1CAM to further investigate this altered expression of cell adhesion molecules at the protein level. In comparison to adherent cells, OVCAR8 spheroids had increased fibronectin expression with multiple isoforms being detected (Figure 4E). Multiple fibronectin isoforms have been attributed to an up-regulation of proteinase activity [36,37], including matrix metalloproteinase-2 (MMP-2), which is up-regulated and cleaves fibronectin to enhance binding to its integrin receptors [14]. Strikingly, there was no detectable fibronectin in OVCAR8-*NUAK1*KO spheroids. In contrast, HEYA8 + NUAK1 cells and spheroids displayed a dramatic increase in fibronectin expression compared to HEYA8 empty-vector control cells and spheroids (Figure 4E). Similar to fibronectin, L1CAM expression was decreased in OVCAR8−*NUAK1*KO in comparison with OVCAR8 adherent cells and spheroids, yet was markedly increased in HEYA8 + NUAK1 spheroids relative to HEYA8 (Figure 4E). These results were further supported by immunofluorescence analysis showing striking fibronectin loss in OVCAR8−*NUAK1*KO spheroids, but dramatically increased expression in HEYA8 + NUAK1 spheroids (Figure 4F). Therefore, our results strongly suggest that NUAK1 regulates fibronectin expression in EOC spheroids.

We treated spheroids with exogenous plasma fibronectin (pFN) to determine whether NUAK1 regulation of fibronectin impacts spheroid integrity. Upon the initiation of spheroid culture, pFN at 5 µg/mL was supplemented to OVCAR8 and OVCAR8-*NUAK1*KO cells. After the same time period as in Figure 3C,D, OVCAR8−*NUAK1*KO spheroids appeared densely packed without protruding peripheral cells, as seen in untreated *NUAK1*KO counterparts (Figure 4G). Indeed, we found that exogenous fibronectin treatment of OVCAR8−*NUAK1*KO spheroids significantly increased the circularity index as compared with untreated OVCAR8−*NUAK1*KO spheroids (Figure 4G). In contrast, we observed no difference in the ability of individual OVCAR8−*NUAK1*KO cells to attach to fibronectin-coated plates as compared with OVCAR8 parental cells (data not shown) [38]. Thus, NUAK1 is required to promote spheroid integrity, likely through its regulation of fibronectin expression and resultant deposition.

### 2.5. NUAK1 Loss in OVCAR8 Cells Extends Survival of Xenografted Mice

**Figure 5:**
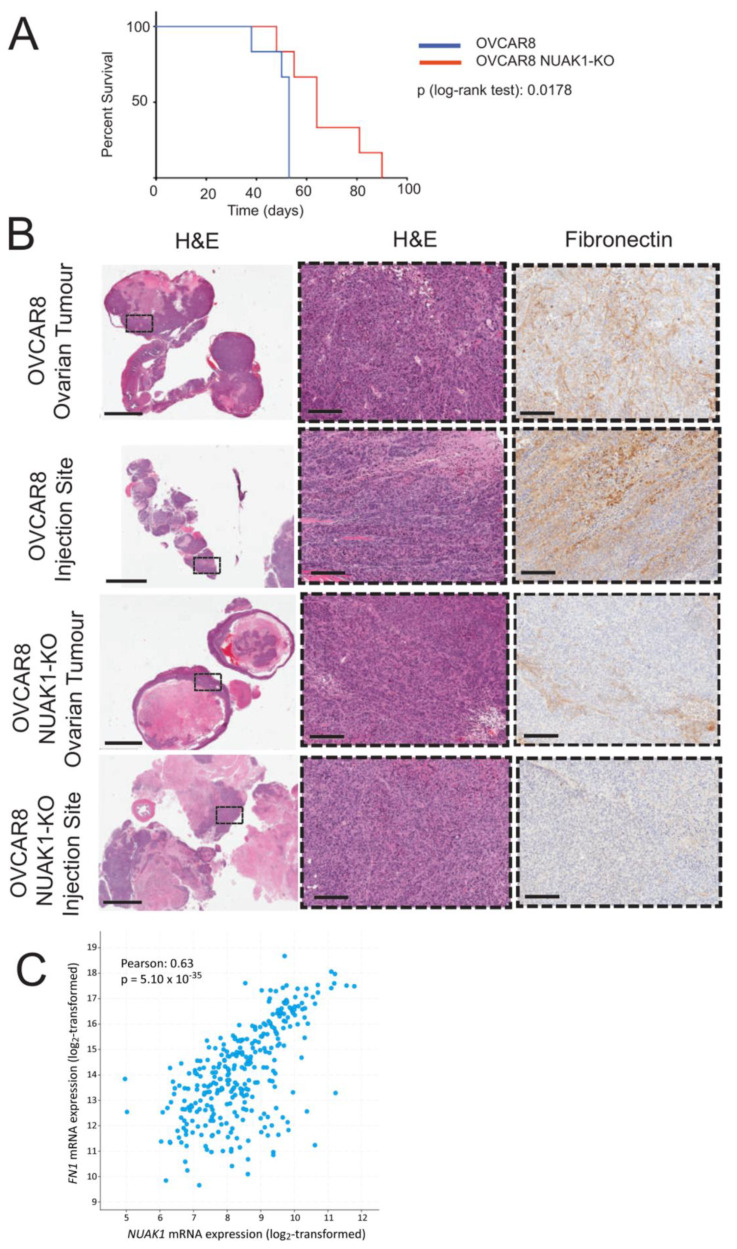
NUAK1 loss in OVCAR8 cells extends survival in xenografted mice. (**A**) Survival analysis for OVCAR8-*NUAK1*KO and OVCAR8 xenografts into female NOD/SCID mice (n = 6). Log-rank test was performed to compare OVCAR8 and OVCAR8–*NUAK1*KO curves. (**B**) Histological analysis of xenografted tumours. Serial sections were stained with hematoxylin and eosin (H&E) or immune-stained for fibronectin as indicated. Black boxes in the low-magnification H&E images encompass an area of interest represented in the high-magnification images. Scale bars represent 4 mm and 200 µm, respectively. (**C**) Correlation analysis between *NUAK1* and *FN1* mRNA expression (log_2_-transformed) in the TCGA Ovarian Serous Cystadenocarcinoma Firehose Legacy dataset (cBioPortal). Pearson correlation coefficient and p value are displayed.

The findings from our in vitro data suggest that NUAK1 increases EOC cell adhesion and enhances spheroid integrity. Since spheroids have a critical role in EOC metastasis, we investigated the effect of NUAK1 loss in a xenograft mouse model of intraperitoneal metastasis. We found that survival was significantly increased in host female mice that were injected i.p. with OVCAR8−*NUAK1*KO cells as compared with OVCAR8 cells (Figure 5A), with a median survival increase of 20.8% (p_log-rank test_ = 0.0178).

We assessed fibronectin directly in tumour xenografts since our in vitro results indicated that NUAK1 promotes spheroid formation through fibronectin expression and deposition. We observed decreased fibronectin immunostaining in OVCAR8−*NUAK1*KO tumours when compared to OVCAR8 controls (Figure 5B). Lastly, we assessed the association between NUAK1 and fibronectin expression in patient tumours. Using The Cancer Genome Atlas (TCGA) ovarian serous cystadenocarcinoma dataset, we found a significant positive correlation (Pearson correlation: 0.63, *p* = 5.10 × 10^−35^) between *NUAK1* and *FN1* mRNA expression in tumours from EOC patients (Figure 5C). Overall, our results indicate that NUAK1 is required for efficient EOC metastasis, likely via its regulation of fibronectin expression during the spread of disease.

## 3. Discussion

Advanced-stage EOC is commonly characterized by malignant ascites containing spheroids, which are a key mediator of intraperitoneal metastasis and facilitate chemoresistance [4,9]. Our group previously reported that the master kinase LKB1 is required for efficient EOC metastasis [15,16]. We completed a multiplex inhibitor bead-mass spectrometry analysis and identified NUAK1 as a top candidate substrate to elucidate the downstream target eliciting the pro-metastatic function of LKB1. NUAK1 is differentially expressed in quiescent spheroids when compared to proliferative monolayer cells and this is regulated by lysosome degradation and the UPS. NUAK1 increases EOC cell adhesion and promotes spheroid integrity via fibronectin expression and subsequent deposition, and this coordinate expression is also seen in human serous tumours. Finally, NUAK1 loss in EOC cells extends xenograft host survival, and the resultant tumours also lack fibronectin. Altogether, we propose that the LKB1 target NUAK1 has metastasis-promoting functions by facilitating spheroid integrity through its regulation of fibronectin production.

LKB1 is known as a master kinase, since it acts through AMPK and twelve additional related kinases called the ARKs to affect cell polarity, metabolism, and growth [17,19]. While LKB1 is commonly regarded as a tumour suppressor, there is growing evidence implicating it as also having pro-metastatic functions [17,39]. We have previously demonstrated that LKB1 is required for EOC spheroid viability in vitro and metastasis in vivo [15,16], and herein we observed that the total LKB1 protein expression increases during late spheroid formation supporting its role in EOC metastasis. Since LKB1 serves a broad range of necessary functions, it is critical to elucidate which of its downstream targets may be more precise therapeutic targets. The most common substrate of LKB1 is AMPK; however, we have evidence that LKB1 elicits its pro-metastatic actions in an AMPK-independent manner in EOC [15]. We identified NUAK1 as the most likely LKB1 target to further investigate in our in vitro metastasis model system while using an unbiased mass spectrometry approach to survey active kinases. Indeed, we demonstrate that LKB1 controls NUAK1 expression and phosphorylation, ultimately affecting NUAK1 stability in EOC cells and spheroids. Previous studies have shown that the UPS plays a role in regulating NUAK1 expression and stability. *NUAK1* can be phosphorylated by cyclin-dependent kinases and polo-like kinases, which leads to SCF E3 ligase-mediated NUAK1 polyubiquitination and subsequent degradation [29]. NUAK1 is ubiquitinated by unique Lys29 and Lys33 linkages, which block phosphorylation and activation by LKB1 [30]. However, USP9X binds to NUAK1 to cleave the polyubiquitin modifications, thereby facilitating LKB1-mediated phosphorylation. In our report, we show that USP9X is required for maintaining NUAK1 expression in EOC. While the UPS is the primary degradation pathway for short-lived and small proteins, the autophagy-lysosome pathway is another degradation system in eukaryotic cells that is responsible for the clearance of damaged proteins, as well as a stress response during nutrient deprivation and hypoxia [40]. Indeed, our group and others have shown that autophagy is activated in EOC, in which spheroids activate autophagy as compared with adherent cells [41,42,43,44]. Our results while using the broad-acting lysosomotropic agent chloroquine to block the late-stages of autophagy indicate that NUAK1 might be degraded by autophagy-lysosome mechanisms, perhaps in a more general way during late spheroid formation. Taken together, NUAK1 is an LKB1 target in EOC to control its differential expression and stability between proliferative adherent cells and quiescent spheroids.

Multiple studies have provided evidence that NUAK1 can have tumour-promoting functions. In human hepatoma cells, NUAK1 blocks programmed cell death by inhibiting caspase 8 [45]. In addition, NUAK1 can induce S-phase to promote cell proliferation [29]. Cancer cell survival is also promoted by NUAK1 through altered metabolic homeostasis [46]. In MYC-overexpressing tumours, NUAK1 reduces metabolic stress by inhibiting mTORC1 and sustaining glutamine metabolism. Relevant to our own findings, an elevated NUAK1 expression correlates with poor prognosis in serous EOC patients [24]. The molecular basis underlying this association with poor prognosis in EOC had not previously been elucidated; however, we show that NUAK1 ablation in EOC cells extends xenografted host survival. Moreover, NUAK1 promotes EOC cell adhesion and spheroid formation, which are essential mediators of intraperitoneal metastasis.

During EOC metastasis, cell adhesion is critical, as cancer cells aggregate through cell–cell and cell–ECM interactions to form spheroids and adhere to new sites [9]. NUAK1 has been documented to play a role in cell adhesion through EMT and invasion. For example, NUAK1 increases EMT and cell migration by inhibiting miR-1181 expression in EOC cells [23]. NUAK1 overexpression in a pancreatic cancer mouse model increased metastasis [47]. NUAK1 can promote cell detachment by regulating the myosin phosphatase complex in HEK293 and MEF cells [22]. Moreover, NUAK1 loss reduces tumour-initiating capacity in colon cancer spheroids [21]. However, in our study we show that NUAK1 loss impairs EOC single cell adhesion to tissue-culture substratum as well as in spheroid formation. We propose that NUAK1 controls the expression of important adhesion molecules and ECM substrates required for the initial steps of spheroid formation. This would explain why NUAK1 protein and phosphorylated-LKB1 are higher at early steps of spheroid formation yet decrease over time. Indeed, the top gene expression signatures altered at 24 h in OVCAR8-*NUAK1*KO spheroids were related to cell attachment. Interestingly, a previous study also observed several cell adhesion pathways affected by NUAK1 using gene ontology analysis yet were not mechanistically pursued [21]. Herein, *FN1* was the most differentially expressed gene in spheroids due to NUAK1 loss. Importantly, we were able to rescue the defect in spheroid integrity due to NUAK1 loss by the addition of soluble fibronectin. Additionally, we showed that xenografted tumours lacking NUAK1 had decreased fibronectin expression, and this was complemented by a strong positive correlation between *NUAK1* and *FN1* in serous EOC tumours from patients. Altogether, we have elucidated a novel mechanism of NUAK1 in promoting EOC cell adhesion and spheroid compaction through fibronectin expression and matrix production.

Beyond just fibronectin, our transcriptome analysis indicated additional genes within a cell attachment signature potentially affected by NUAK1, which included *L1CAM, ITGβ8, ITGβ5, THBS1*, and *F11R*. This suggests that NUAK1 might regulate a network of adhesion molecules within EOC spheroids. A previous study using OVCAR5 cells showed that fibronectin mediates spheroid formation through its canonical α5β1 integrins [11]. Interestingly, these specific integrins were not altered in our system, but rather the *ITGβ5* and *ITGβ8* were significantly reduced due to NUAK1 loss. One study has shown that β8-integrin might interact with fibronectin; however, this was only observed in chick sensory neurons and the alpha integrin was not identified [48]. Interestingly, a common ligand for both β5- and β8-integrins is latency-associated peptide-transforming growth factor beta (LAP-TGFβ) [49], and we have shown that active TGFβ signalling in EOC spheroids is critical for promoting EMT in these structures [50]. Thus, NUAK1 might cross-talk to impact TGF β-mediated EMT in EOC spheroids, as observed in other cancer cell systems [51]; this will be a focus of on-going study by our group.

Another study similarly demonstrated the importance of fibronectin in spheroids generated from fallopian tube secretory epithelial (FTE) cells, which represent the cell-of-origin for high-grade serous EOC [52,53]. FTE cells with *TP53* mutations have an increased propensity to aggregate into spheroids due to autocrine fibronectin deposition [54]. Others have observed that L1CAM acts to up-regulate fibronectin expression in order to facilitate spheroid formation [35]. In our study, we similarly show a strong coordinate regulation between L1CAM and fibronectin, implying that NUAK1 might be an important upstream signal controlling their expression together to promote spheroid formation and ultimately EOC metastatic potential.

## 4. Materials and Methods

### 4.1. Antibodies and Reagents

Antibodies against NUAK1 (#4458S), LKB1 (#3050S), p-LKB1-Ser428 (#3482S), LC3B (#2775), and c-myc (#5605) were obtained from Cell Signaling Technology (Danvers, MA, USA). Anti-tubulin antibody (#T5168), Anti-actin antibody (#A2066), anti-rabbit FITC secondary antibody (# F9887), HRP-conjugated antibodies against mouse IgG (NA931V), and rabbit IgG (NA934V), and 4′,6-diamidino-2-phenylindole were purchased from Sigma (St. Lewis, MO, USA). Chloroquine (#C-6628), MG132 (#M8699), and methylcellulose (#M0512) were also obtained from Sigma. Anti-fibronectin (#ab2413) was purchased from Abcam (Cambridge, MA, USA). Antibody against L1CAM (#SIG-3911) was obtained from Biolegend (San Diego, CA, USA). Anti-USP9X (A301-350A) was purchased from Bethyl Laboratories (Montgomery, TX, USA). Alexa Fluor phalloidin and plasma human fibronectin (#PHE0023) was obtained from Thermo Fisher Scientific (Waltham, MA, USA). WZ4003 (#5177) was purchased from Tocris Bioscience.

### 4.2. Cell Culture and Treatments

OVCAR8 (ATCC, Manassas, VA, USA) and HEYA8 (ATCC) cells were cultured in RPMI-1640 (Wisent, St. Bruno, QC, Canada). The OVCAR5 cells (ATCC) were cultured in DMEM/F12 (Life Technologies, Carlsbad, CA, USA). Although one report suggests OVCAR5 cells may be of gastrointestinal origin [55], this cell line was isolated from the ascites of an EOC patient and it maintains the ability to form i.p xenografts with HGSOC histology [28]; therefore, we have included it our study. Early passage ascites-derived cell lines (iOvCa147, iOvCa198, iOvCa247) were generated based on a protocol that was previously described by us and cultured in DMEM/F12 [56]. Generation of OVCAR8-*STK11*KO cells was described previously [15], and cultured the same as OVCAR8 cells. The growth media was supplemented with 10% fetal bovine serum (Wisent) for all cell lines. The cells were grown in a humidified incubator at 37 °C with 5% CO_2_.

Adherent cells were maintained on tissue cultured-treated polystyrene (Sarstedt, Newton, NC, USA). Spheroids were formed by maintaining cells on Ultra-Low Attachment (ULA) cluster plates (Corning, NY, USA), which have a hydrophilic and neutral coating to prevent cell attachment, as described previously [7,16,41]. For specific experiments, day-3 adherent cells and spheroids were treated with 0.1% DMSO or MG132 (10 μM) for 8 h; cells were treated with chloroquine (25 μM) for 8 h or left untreated.

### 4.3. Generation of OVCAR8-NUAK1KO Cells

Two independent 20-nucleotide guide sequences targeting the *NUAK1* gene 5′-GTGGC GGGGG ACCGC CCCGA-3′ (site 1) and GGGTC TCCTG CAGCT CGTAG CGG-3′ (site 2) were selected while using CRISPR Design Tool (http://tools.genome-engineering.org). Complementary oligonucleotides 5′-CACCG TCGGG GCGGT CCCCC GCCAC-3′and 5′-CACCG GGGTC TCCTG CAGCT CGTAG-3′ for site 1 and 5′-CACCG GGGTC TCCTG CAGCT CGTAG-3′ and 5′-AAACC TACGA GCTGC AGGAG ACCCC-3′ for site 2 (Sigma-Genosys) were annealed and ligated into the *Bbs*I-digested restriction endonuclease site of pSpCas9(BB)-2A-Puro plasmid [57] (gift from Dr. F. Dick, Western University) to generate the pSpCas9-sg*NUAK1*-1 and -2 plasmids. Cells were seeded at 200,000 cells/well into 6-well plates and transfected with 0.5 µg each of pSpCas9-sgNUAK1 plasmids while using LipofectAMINE 2000 (Invitrogen) according to the manufacturer’s instructions. Media containing 1 μg/mL puromycin was replaced the following day, and cells were treated for one day. After growth recovery, the cells were trypsinized, counted, and seeded into 96-well plates to perform limiting dilution subcloning of *NUAK1*-knockout cells. Single colonies were expanded for protein isolation and the confirmation of NUAK1 loss by western blotting. Three clones lacking NUAK1 protein expression were identified and verified by genomic DNA isolation and Sanger sequencing (London Regional Genomics Centre, Robarts Research Institute).

### 4.4. Generation of HEYA8 and OVCAR3 NUAK1 Overexpressing Cells

HeyA8 and OVCAR3 cells were transfected with pPHAGE C-TAP-NUAK1 (HsCD00462473; Harvard PlasmID Database) while using Lipofectamine 2000 (Invitrogen) according to manufacturer’s instructions. Forty-eight hours post-transfection, 1 µg/mL puromycin (Sigma) treatment was started, and then cells were re-plated into 10-cm dishes and selection was continued until colony formation. Colonies were picked and expanded prior to screening for NUAK1 expression by western blot using lysates from parental cell lines for comparison. Empty vector control (EVC) cells were generated while using the pPHAGE C-TAP plasmid for both cell lines and represent four pooled clones of each line subjected to the same puromycin selection process.

### 4.5. Spheroid Live-Cell Microscopy

The cells were seeded in a 24-well ULA dish at 1000 cells per well and cultured in complete media and methylcellulose [34]. For the fibronectin rescue experiment, 5 µg/mL plasma fibronectin was included in the media. After 11 days, spheroids were washed with PBS and transferred to a new ULA dish with only complete media. The images were immediately taken with the Leica DMI 4000B inverted microscope using the Leica Application Suite version 4.4 software. The circularity of spheroids was analysed through Fiji Is Just ImageJ (Fiji) (http://fiji.sc), and 7–10 individual spheroids were analysed per experiment. A pixel size range of 1000–infinity and a circularity range of 0–1.0 was used for all images.

OVCAR8 and OVCAR8-*NUAK1*KO cells stably-transduced with lentivirus expressing NucLightGFP (Sartorius) were seeded at 5000 cells per well in 96-well round-bottom ULA cluster plates (Corning). The spheroids were imaged using the IncuCyte Zoom (Sartorius) every 3 h for up to 17 days.

### 4.6. Cell Adhesion Assay

The cells were seeded in a 24-well adherent dish at 200,000 cells per well. At specific time points for each EOC cell line (15 min for HEYA8 cells, 2 h for OVCAR3 cells, and 4 h for OVCAR8 cells), non-adherent cells were aspirated, the plate was washed with PBS, and adherent cells were counted by Trypan Blue Exclusion cell counting in order to quantify single viable cell adhesion.

### 4.7. Immunofluorescence

The spheroids were embedded in cryo-matrix (Thermo Fisher) and sectioned at 5 µm with Shandon cryostat microtome. Cryosections were fixed (10% formalin solution), permeabilized (0.1% Triton X-100 in PBS), and then blocked (5% BSA in 0.1% Triton X-100). After overnight incubation with anti-fibronectin antibody (1:100; #ab2413), sections were washed with PBS and then incubated for 1 h with anti-rabbit FITC secondary antibody (1:300; # F9887). For counterstaining, sections were incubated for 1 h with Alexa Fluor phalloidin (1:1000), followed by incubation for 1 h with 4′,6-diamidino-2-phenylindole (1:1000). Sections were mounted on coverslips with Vectashield (Vector Laboratories, Burlingame, CA, USA). Fluorescence images were captured while using Olympus AX70 upright microscope and ImagePro image capture software.

### 4.8. Multiplexed Inhibitor Bead Chromatography

Lysates were collected from OVCAR8 parental and OVCAR8-*STK11*KO cells that were cultured as adherent cells and spheroids. Multiplexed Inhibitor Bead (MIB) Chromatography was performed, as described below.

Broad spectrum Type I kinase inhibitors (CTx0294885, VI-16832, PP58, Purvalanol B, UNC-2147A, UNC-8088A) were custom-synthesized with hydrocarbon linkers and terminal amine groups and covalently attached to ECH-activated Sepharose beads, as previously described [58], to form the multiplexed inhibitor beads (gift from Gary Johnson, UNC). The enrichment of kinases from OVCAR8 cells by MIB chromatography was adapted from [25]. The cell pellets were lysed in MIB lysis buffer (50 mM HEPES pH 7.5, 150 mM NaCl, 0.5% Triton X-100, 1 mM EDTA, 1 mM EGTA freshly supplemented with 10 mM NaF, 2.5 mM NaVO_4_, protease inhibitor cocktail (Sigma–Aldrich, Catalog #P8340), phosphatase inhibitor cocktail 2 (Sigma–Aldrich, Catalog #P5726), and phosphatase inhibitor cocktail 3 (Sigma–Aldrich, Catalog #P0044)) while on ice for 20 min. The lysate was homogenized with an 18-gauge syringe needle and centrifuged at 20,800× *g* for 15 min at 4 °C. The total protein amount in the supernatant was quantified using the Bio-Rad protein assay (Catalog #5000006) according to the manufacturer’s instructions. The supernatant was brought to 1 M·NaCl and 4 mg of total protein was loaded on a column (Bio-Rad, Catalog #731–1550) of 100 µL packed ECH sepharose 4B (GE Healthcare, Catalog # 17057101), pre-equilibrated with 2 mL of high salt Buffer B (50 mM HEPES pH 7.5, 1 M·NaCl, 0.5% Triton X-100, 1 mM·EDTA, 1 mM·EGTA). The flowthrough was transferred to a column consisting of layered MIBs (50 µL each of a 50% slurry of CTx0294885, VI-16832, PP58, Purvalanol B, UNC-2147A, and UNC-8088A) pre-equilibrated with 2 mL of Buffer B. The flowthrough was reapplied to the column and the column was then washed with 5 mL of Buffer B and 5 mL of 50 mM ammonium bicarbonate (ABC). After washing, the beads were transferred to 1.5 mL centrifuge tubes in 1 mL of 50 mM ABC and then washed twice more in 1 mL of 50 mM ABC. The samples were digested overnight at 37 °C with 1 µg trypsin and LysC (Promega, Catalog # V5073). The supernatant was collected and the samples were reduced in 5 mM DTT at 53 °C for 30 min., cooled to room temperature, and then alkylated in 10 mM iodoacetamide in the dark at RT for 45 min. Trypsin (Sigma–Aldrich, Catalog # T6567) was added and the samples were incubated at 37 °C for 4 h. Formic Acid was added to a final concentration of 2%, and the samples were dried by speed vac.

### 4.9. Mass Spectrometry Analysis

The digested peptides were dissolved in 5% Formic Acid and then transferred to autosampler vials for analysis by nano-LC-MS/MS while using a SCIEX 5600 TripleTOF mass spectrometer and an Eksigent Ultra nanoHPLC. The samples were loaded onto a home-packed emitter tip column (15 cm × 75 um; 3 µm·C18, Reprosil, Dr. Maisch). After sample loading, a linear gradient from 2% acetonitrile to 35% acetonitrile over 90 min. at 200 nL/min was used to elute all peptides. A further increase to 80% acetonitrile from 90–95 min., hold from 95–105 min. at 80% acetonitrile, and return to 2% acetonitrile from 105 min. to 120 min. was used to ensure full peptide elution. During peptide elution, data were acquired on the mass spectrometer in data-independent acquisition (DIA) mode. Cycle time was 3.5 s, consisting of a 250 ms MS1 scan (400–1250 Da) and 34 × 25 Da SWATH windows covering the range of 400–1250 m/z.

### 4.10. Mass Spectrometry Data Analysis

All of the raw MS files were saved in our local interaction proteomics LIMS, ProHits [59]. The WIFF raw files were converted into mzXML format while using the SCIEX converter through the Proteowizard module implemented within ProHits. The mzXML files were processed by the signal extraction (SE) module of DIA-Umpire [60] (version 2.0) to generate pseudo MS/MS spectra for data base searches. The following parameters were used: isolation window (fixed, 25 Da), fragment grouping (RPmax 25, RFmax 300, correlation threshold 0.2, delta apex 0.6, RT overlap 0.3), signal extraction parameters [mass tolerance (30 ppm MS1, 40 ppm MS2), signal to noise (2 for MS1 and MS2), minimum intensity threshold (1 for MS1, 0.1 for MS2), charge state range (2+ to 4+ for MS1 and MS2), maximum curve in RT range (1.5), and resolution (17,000)]. The files were searched using X! tandem (version Jackhammer, 2013.06.15.1) and Comet (version 2014.02 revision 2) using the following parameters: allow tryptic peptides only, carbamidomethylation on cysteine as a fixed modification, and deamidation on asparagine and glutamine, oxidation on methionine, and phosphorylation on serine, threonine and tyrosine as variable modifications. Additional Comet parameters were two missed cleavages, monisotopic parent and fragment mass, 35 ppm peptide mass tolerance, 2+ to 4+ precursor charge state, fragment ion binding (1.005 amu with 0.4 offset). Additional X! tandem parameters were: one missed cleavage, 50 ppm parent mass error, 40 ppm fragment mass error, monoisotopic fragment, and 4+ maximum parent charge. The searched database contained the human and adenovirus complements of the RefSeq protein database (version 57) supplemented with ‘‘common contaminants’’ from the Max Planck Institute (http://141.61.102.106:8080/share.cgi?ssid=0f2gfuB) and the Global Proteome Machine (GPM; http://www.thegpm.org/crap/index.html), as well as sequences from common fusion proteins and epitope tags. The sequence database consisted of forward and reversed sequences; in total, 72,226 sequences were searched. The resulting Comet and X! tandem search results were individually processed by PeptideProphet [61], and the peptides were assembled into proteins while using parsimony rules that were first described in ProteinProphet [62] into a final iProphet [63] protein output using the Trans-Proteomic Pipeline (TPP; Linux version, v4.7, Polar Vortex rev 1, Build 201410231114). The TPP options were as follows: general options were -p0.05 -x20 -PPM - d’’DECOY,’’ iProphet options were pPRIME and PeptideProphet options were pPAEd. Parameters for DIA-Umpire Quant (version 2.0) were peptide FDR (0.05), protein FDR (0.05), probability threshold (0.9), filter weight (group), minimum weight (0.9), top number of fragments (20), top number peptides (20), and frequency (0). mapDIA analysis [64] (version 2.3.3) was then performed on the DIA-Umpire results for intensity normalization, selection of fragments and peptides, and the determination of significantly changed proteins. For this, only peptides that were unique at the gene level were considered. mapDIA used the following parameters: impute (group 0.9, missing values are assigned 0.9 of the smallest value of the group in the row; if none above zero, then 0.9 the smallest value of the column), experimental design (replicate), normalization (retention time normalized with standard deviation of 10, rounded to two decimal places (RT 10 2)), standard deviation factor (SDF) filter (2), minimum correlation (2), minimum observation (2), minimum fragments per peptide (3), maximum fragments per peptide (5), minimum peptides per protein (2), maximum peptides per protein (infinity), minimum proportion of differentially expressed proteins (0.01), and maximum proportion of differentially expressed proteins (0.99). All of the MS data will be made available through the MassIVE repository at ftp://massive.ucsd.edu/MSV000085252/.

### 4.11. Protein Isolation and Immunoblot Analysis

The cells were collected after washing the plate with PBS and scraping cells in lysis buffer [50 mM HEPES pH 7.4, 150 mM NaCl, 10% glycerol, 1.5 mM MgCl_2_, 1 mM EGTA, 1 mM sodium orthovanadate, 10 mM sodium pyrophosphate, 10 mM NaF, 1% Triton X-100, 1% sodium deoxycholate, 0.1% SDS, 1 mM PMSF. 1X protease inhibitor cocktail (Roche, Laval, QC, Canada), and 225 mM β-glycerophosphate] in order to obtain whole cell lysates from adherent culture. Spheroids were centrifuged at 2400 rpm for 5 min., media was aspirated, and the pellet was washed in ice-cold PBS. After the removal of PBS, lysis buffer was added to the pellet. Protein was isolated and the protein concentration in the supernatant was measured by Bradford assay (Bio-Rad Laboratories, Hercules, CA, USA). The protein was isolated from xenograft tumour samples collected from a previous study [15] by homogenizing flash-frozen tissue in lysis buffer without β-glycerophosphate.

For immunoblot analysis, 30–50 µg of protein was resolved by SDS-PAGE using 6%, 8%, or 12% gels. The proteins were transferred at 100 V for 1 h to a PVDF membrane (Roche), blocked with 5% milk or 5% BSA diluted in TBST (10 mM Tris–HCl, pH 8.0, 150 mM·NaCl and 0.1% Tween-20). The membranes were incubated with primary antibodies overnight at 4 °C. Membranes were incubated for 1 h with peroxidase-conjugated anti-rabbit or anti-mouse antibodies (1:10,000 in 5% BSA/ TBST) and exposed to chemiluminescence reagent to obtain whole cell lysates from adherent culture (Luminata Forte, Millipore, Temecula, CA, USA). The images were captured using the ChemiDoc™ Imaging System (Bio-Rad) and the bands were quantified using Image Lab 4.1 software.

### 4.12. Phostag^TM^ Western Blot

Phostag^TM^ lysis buffer was prepared similar to above; however, EGTA, sodium pyrophosphate, and β-glycerophosphate were excluded from the preparation. PhostagTM gels were prepared while using Phostag^TM^ solution (Wako Chem, Richmond, VA, USA) and 10 mM MnCl_2_, as 8% acrylamide gels, according to manufacturer’s protocol. Electrophoresis was run for ~3 h, after which gels were washed for 10 min. with 1× transfer buffer containing 1 mM EDTA, followed by 10 min. with 1× transfer buffer without EDTA. Wet transfer was run for 1 h to PVDF membranes (Immobilon-P). The membranes were incubated in primary antibody for two days, and imaging was performed as described above.

### 4.13. Quantitative RT-PCR

Total RNA was isolated from adherent cells and spheroids while using the RNeasy Mini Kit (Qiagen, Hilden, Germany). The concentration and purity of the RNA were determined with the ND-1000 spectrophotometer (NanoDrop Technologies, Wilmington, DE, USA). Reverse transcription was performed using High Capacity cDNA Reverse Transcription Kit (*Applied Biosystems*, Foster City, CA, USA). The resulting cDNA was used for quantitative PCR while using Brilliant SYBR Green QPCR Master Mix (Agilent Technologies/Stratagene, Mississauga, ON, Canada) and run on the Quantstudio 3 instrument (Applied Biosystems). Table S1 lists human-specific primer sequences (Sigma). *GAPDH* was used as an internal control, and relative gene expression was quantified using the ∆∆Ct method.

### 4.14. Transcriptome Analysis and GSEA

The RNA was isolated from OVCAR8 and OVCAR8-*NUAK1*KO adherent cells and spheroids, as described above. The RNA was reverse transcribed into cDNA and then labelled with biotin using the Affymetrix Genechip WT pico kit (Thermo Fisher Scientific). The labelled cDNA was hybridized to the Human Clariom S microarray (Thermo Fisher Scientific). After washing, the microarray was scanned while using the Affymetrix Gene Chip Scanner 3000. Data analysis was completed with the Transcriptome Analysis Console software at The Centre for Applied Genomics (TCAG Facility, SickKids Hospital).

Pathway analysis was completed using Gene Set Enrichment Analysis (GSEA) version 3.0 that was developed by the Broad Institute at MIT [65]. The gene list was imported into GSEA without limiting the genes by applying cut-offs. The Hallmark collection of 50 gene sets and the Curated Canonical collection of 1329 gene sets from the Molecular Signatures Database (MSigDB, Broad Institute) were used for the analysis. These gene sets were limited to those with 15 to 500 genes. Permutations were completed 1000 times. Signal-to-noise was calculated and used to rank genes based on their differential expression, where significance was set at *p* < 0.05 and FDR < 0.25.

### 4.15. TCGA Correlation Analysis

Correlation analysis of *NUAK1* and *FN1* mRNA expression (RNA Seq V2 RSEM) was performed while using the Firehose Legacy dataset from The Cancer Genome Atlas (TCGA) Ovarian Serous Cystadenocarcinoma in cBioPortal [66,67].

### 4.16. Xenotransplantation Assays

Female NOD/SCID mice (Charles River, 8–10 weeks old) were intraperitoneally injected with 4 × 10^6^ OVCAR8 or OVCAR8-NUAK1KO cells in 150 µL of sterile PBS. The mice were monitored every day and provided chow (cat. No.2919, Envigo) and water ad libitum. Euthanasia occurred when humane endpoint criteria were met and necropsy was performed as described previously [15]. The Institutional Animal Care and Use Committee of the University of Western Ontario (AUP # 2017-065) approved all of the animal experiments and carried out in accordance with the approved guidelines.

### 4.17. Statistical Analysis

Statistical analysis was completed using GraphPad PRISM 7 (GraphPad Software, San Diego, CA, USA). Analyses were performed while using two-tailed Student’s *t*-test, multiple *t*-test with Bonferroni correction, one-way ANOVA, followed by Dunnett’s *post hoc* test, or two-way ANOVA followed by Tukey’s *post-hoc* test. A *p*-value less than 0.05 was considered to be statistically significant.

## 5. Conclusions

NUAK1 serves as a key LKB1 target in EOC to promote its pro-metastatic functions via cell adhesion and spheroid integrity. We propose that NUAK1 controls fibronectin expression in spheroids, leading to its deposition within the ECM, thereby facilitating EOC metastasis. Since LKB1 serves multiple key functions that may make it unsuitable for targeted inhibition, then the identification of NUAK1 as a key mediator acting immediately downstream could serve as a better drug target. Indeed, there are several small molecule inhibitors directed against NUAK1/2 [68,69] and these could be directly tested, or used as scaffolds for deriving more efficacious agents in the future. Altogether, our latest results suggest that the LKB1 substrate NUAK1 might serve as a novel therapeutic target in advanced-stage EOC.

## Supplementary Materials

The following are available online at https://www.mdpi.com/2072-6694/12/5/1250/s1, Figures S1–S45 represent the whole blot images for Figure 1A,C,D,E, Figure 2A,B,D,E,F, Figure 3A and Figure 4E. Table S1: Primer sequences for qPCR, Table S2: Differentially expressed kinases (FDR < 0.05 from mapDIA) from MIB/MS analysis completed using OVCAR8 parental and OVCAR8 STK11-KO adherent cells, Table S3: Differentially expressed kinases (FDR < 0.05 from mapDIA) from MIB/MS analysis completed using OVCAR8 parental and OVCAR8 STK11-KO spheroids, Table S4: Differentially expressed genes from the Affymetrix Clariom S array using OVCAR8 parental and OVCAR8 NUAK1-KO spheroids, Table S5: Signatures from the GSEA Hallmark database that were enriched in the OVCAR8 parental spheroids compared to NUAK1-KO spheroids, Table S6: Signatures from the Curated Canonical database that were enriched in the OVCAR8 parental spheroids compared to NUAK1-KO spheroids, Table S7: Core enriched gene set from REACTOME_INTEGRIN_CELL_SURFACE_INTERACTION signature.
