# Supplementary material for: A Novel Role for NUAK1 in Promoting Ovarian Cancer Metastasis through Regulation of Fibronectin Production in Spheroids
Source: Cancers (Basel). 2020 May 15;12(5):1250. doi: 10.3390/cancers12051250 (PMC7280971; doi:10.3390/cancers12051250)
Supplement: Supplementary file 1 [file cancers-12-01250-s001.zip › Supplementary Material.docx]

Supplementary Material: A Novel Role for NUAK1 in Promoting Ovarian Cancer Metastasis through Regulation of Fibronectin Production in Spheroids

Jamie Lee Fritz, Olga Collins, Parima Saxena, Adrian Buensuceso, Yudith Ramos Valdes, Kyle E. Francis, Kevin R. Brown, Brett Larsen, Karen Colwill, Anne-Claude Gingras, Robert Rottapel and Trevor G. Shepherd


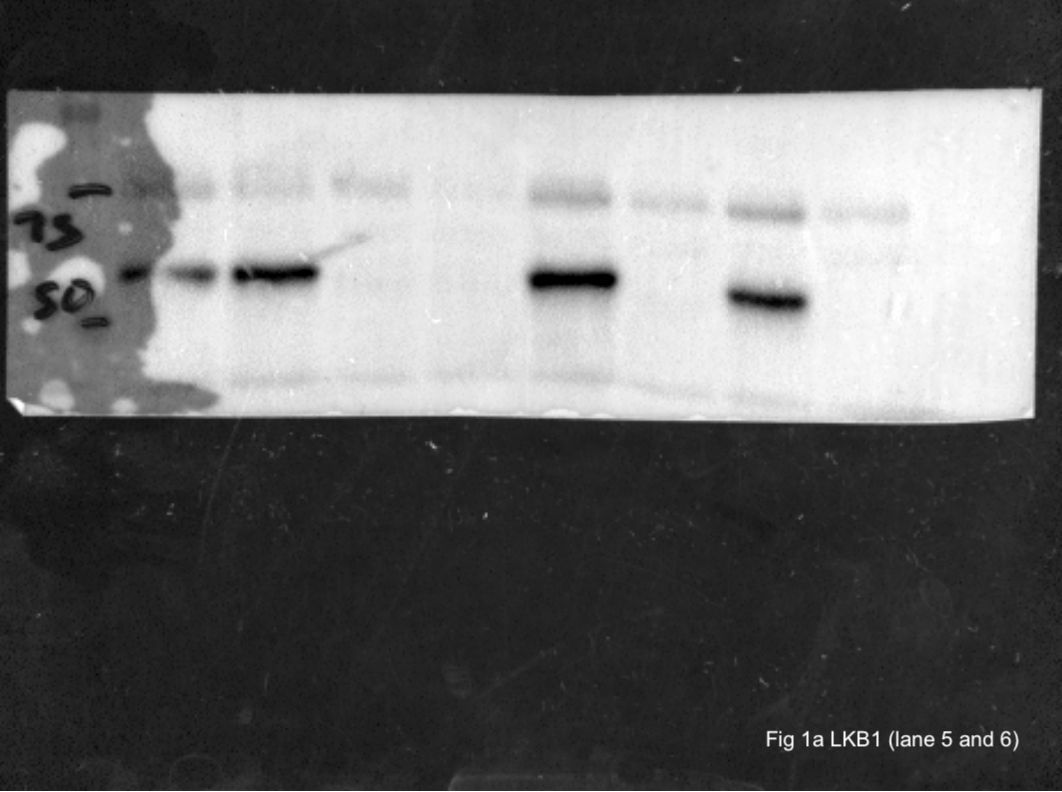


**Figure S1.** Whole blot image of LKB1 (lane 5 and 6) from Figure 1A.


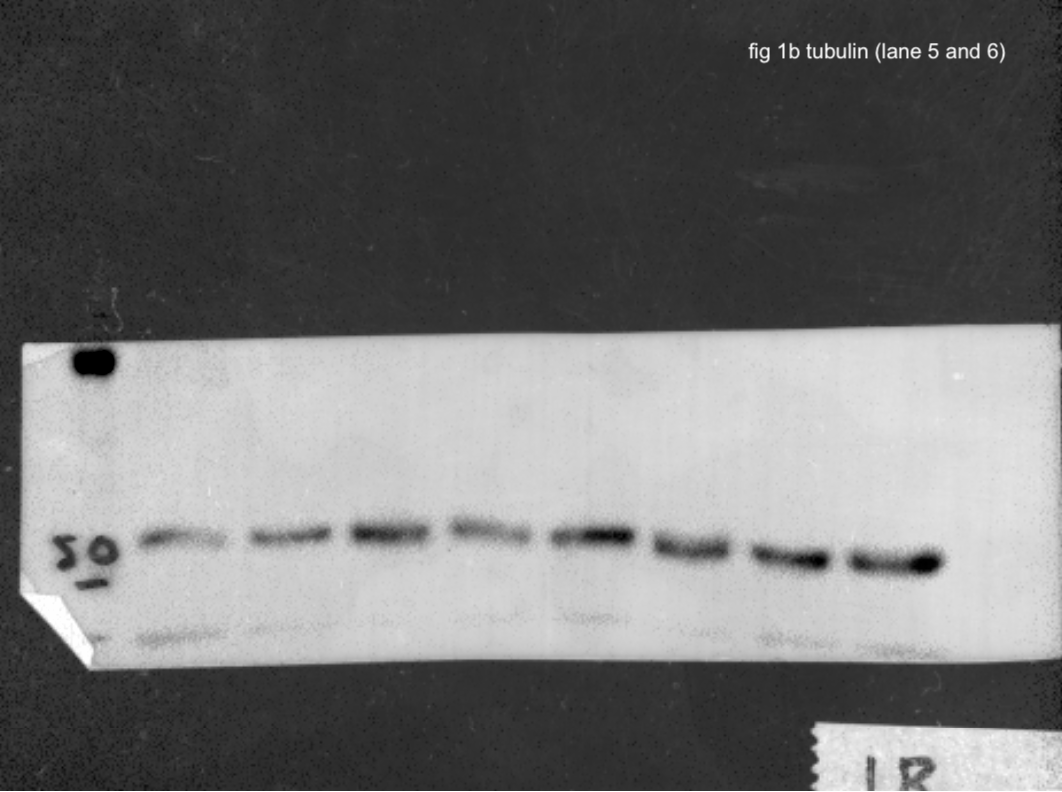


**Figure S2.** Whole blot image of Tubulin (lane 5 and 6) from Figure 1A.


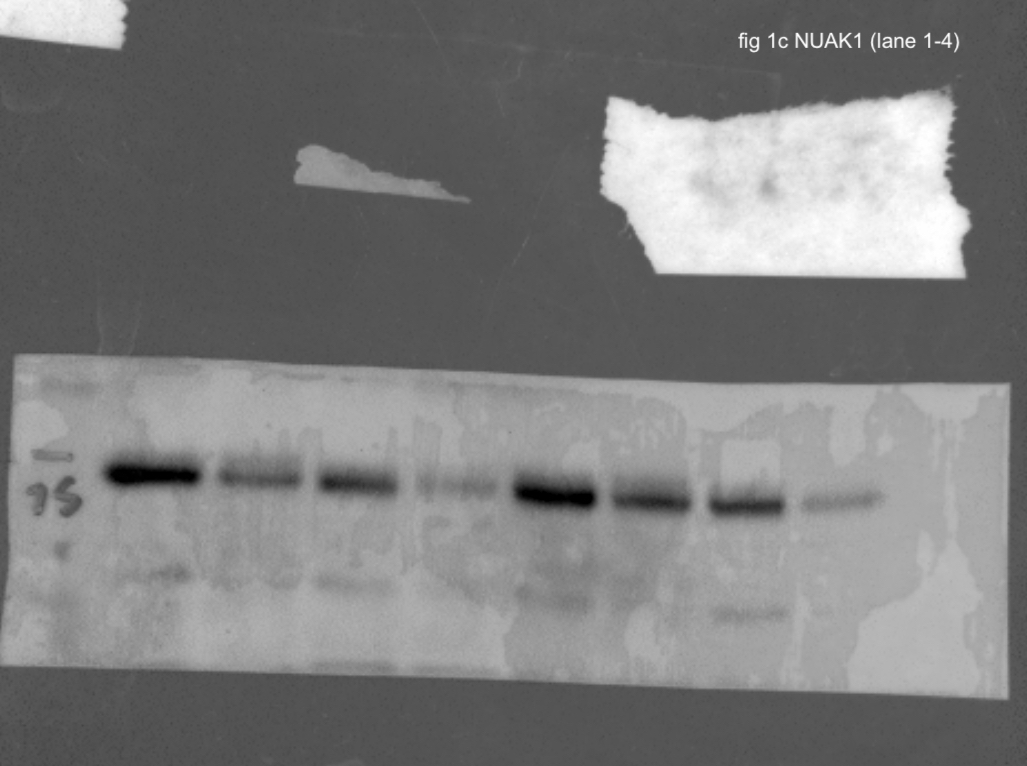


**Figure S3.** Whole blot image of NUAK1 (lane 1–4) from Figure 1C.


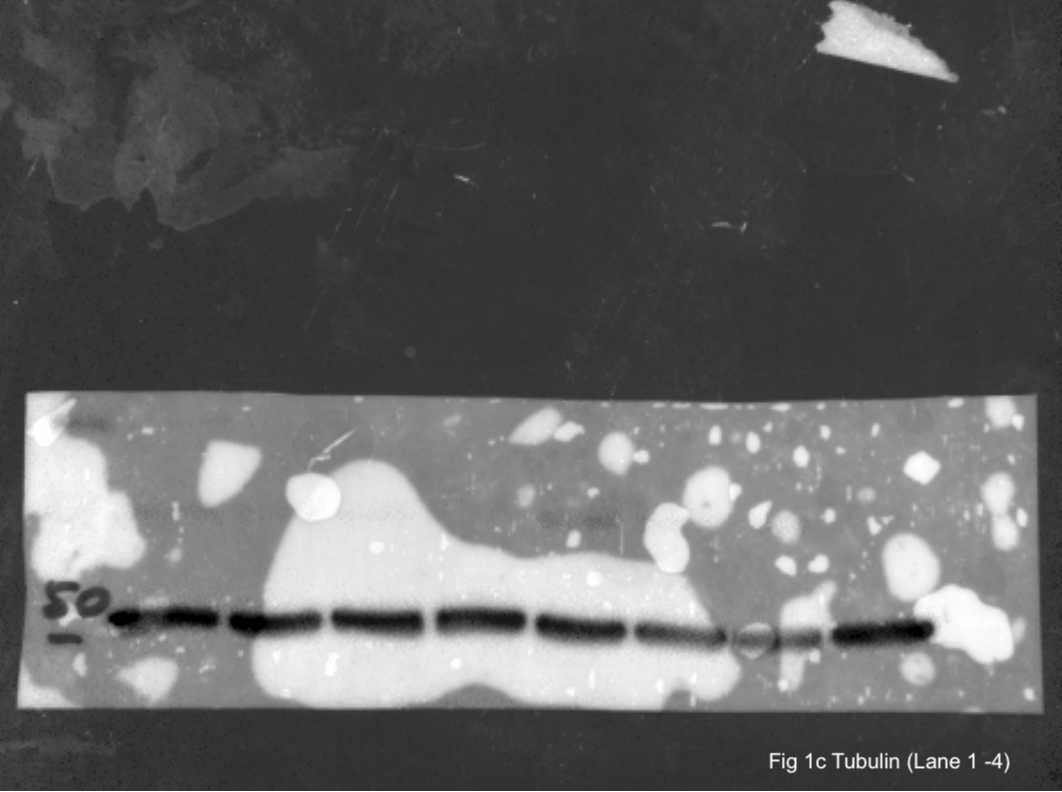


**Figure S4.** Whole blot image of Tubulin (lane 1–4) from Figure 1C.


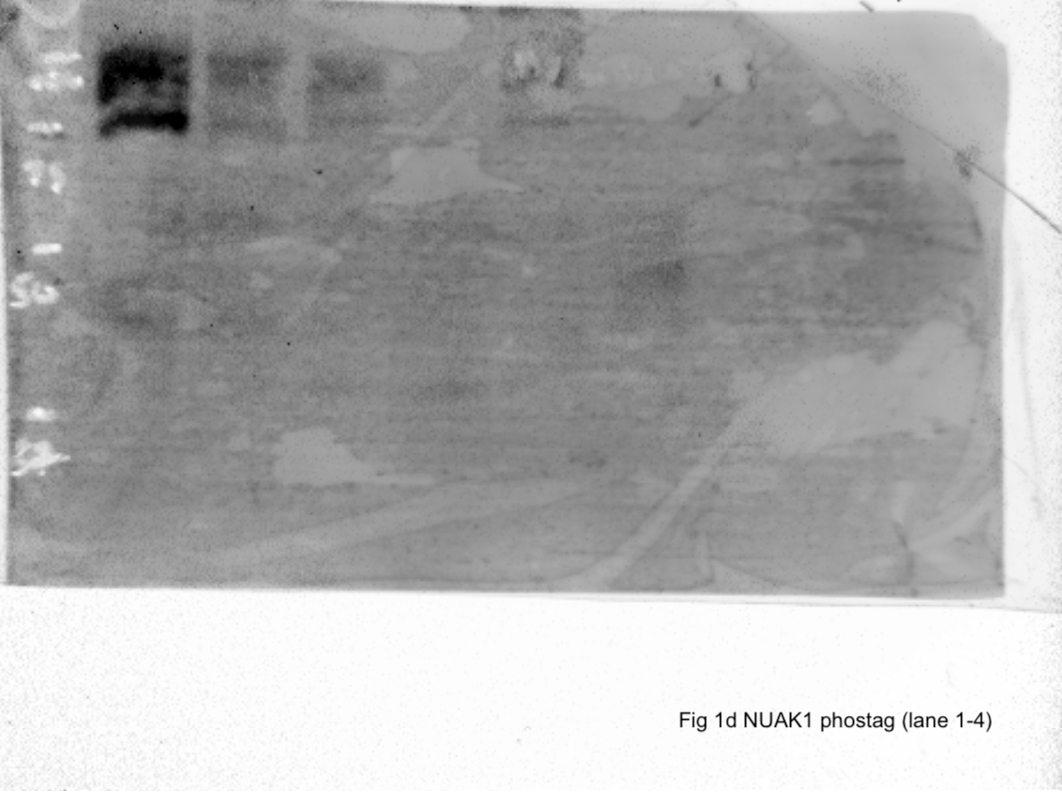


**Figure S5.** Whole blot image of p-NUAK1 (lane 1–4) from Figure 1D.


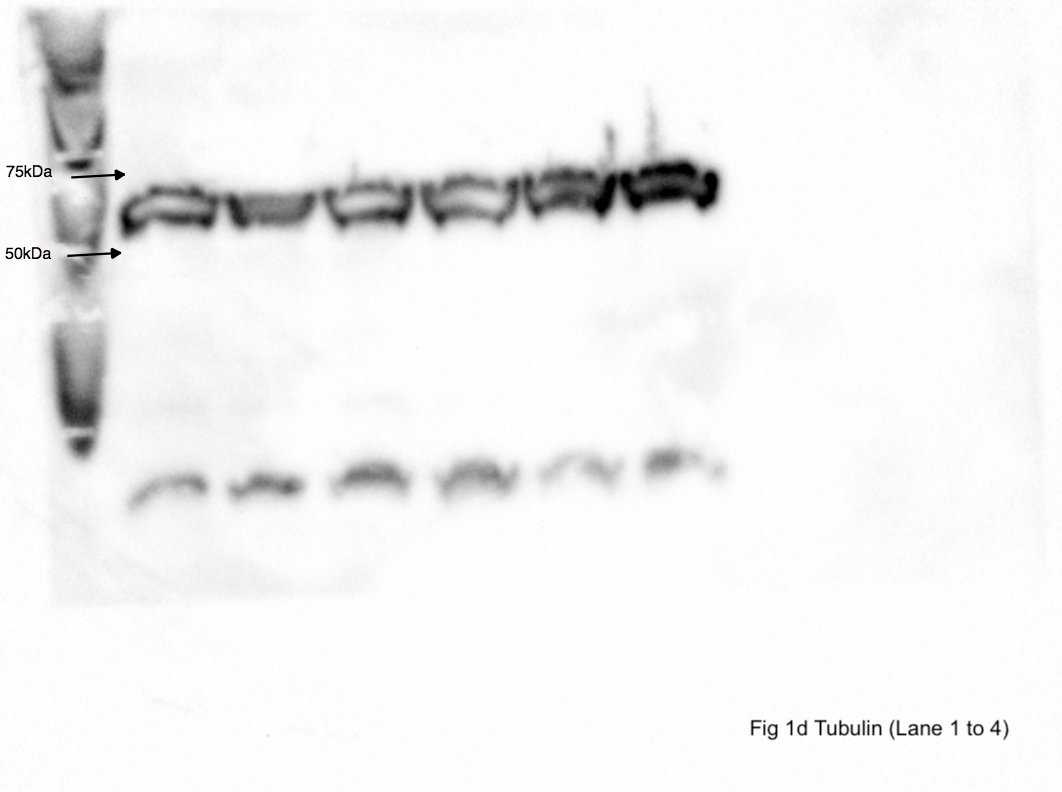


**Figure S6.** Whole blot image of Tubulin (lane 1–4) from Figure 1D.


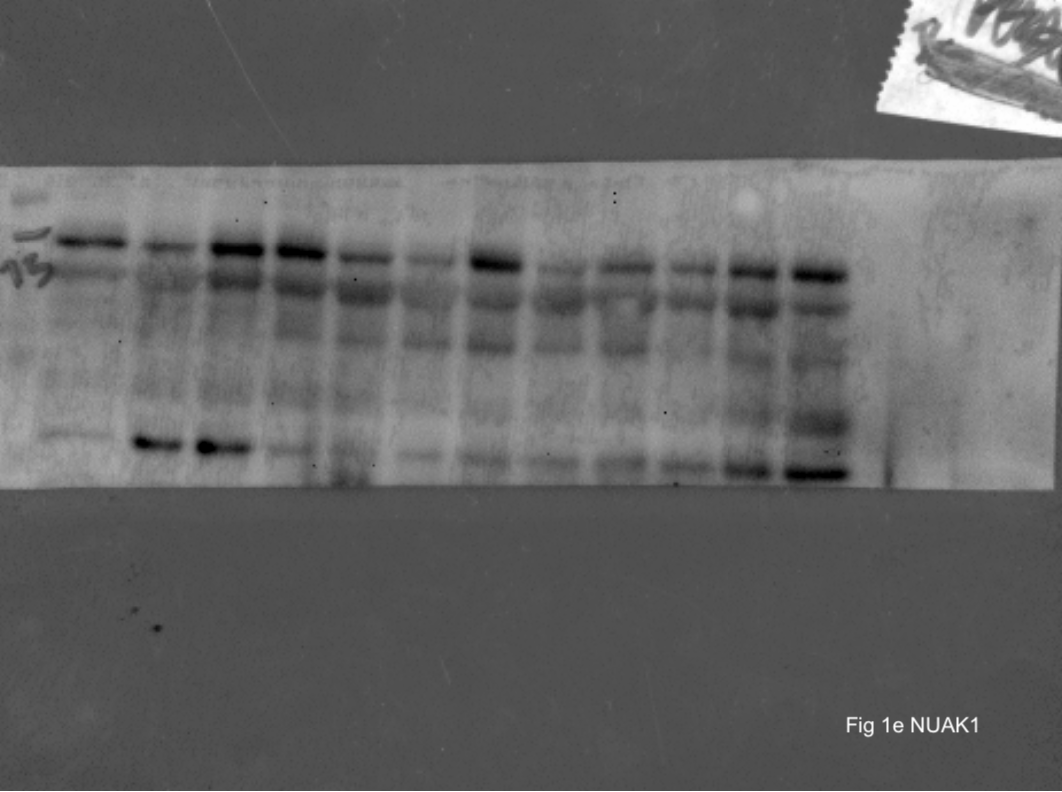


**Figure S7.** Whole blot image of NUAK1 from Figure 1E.


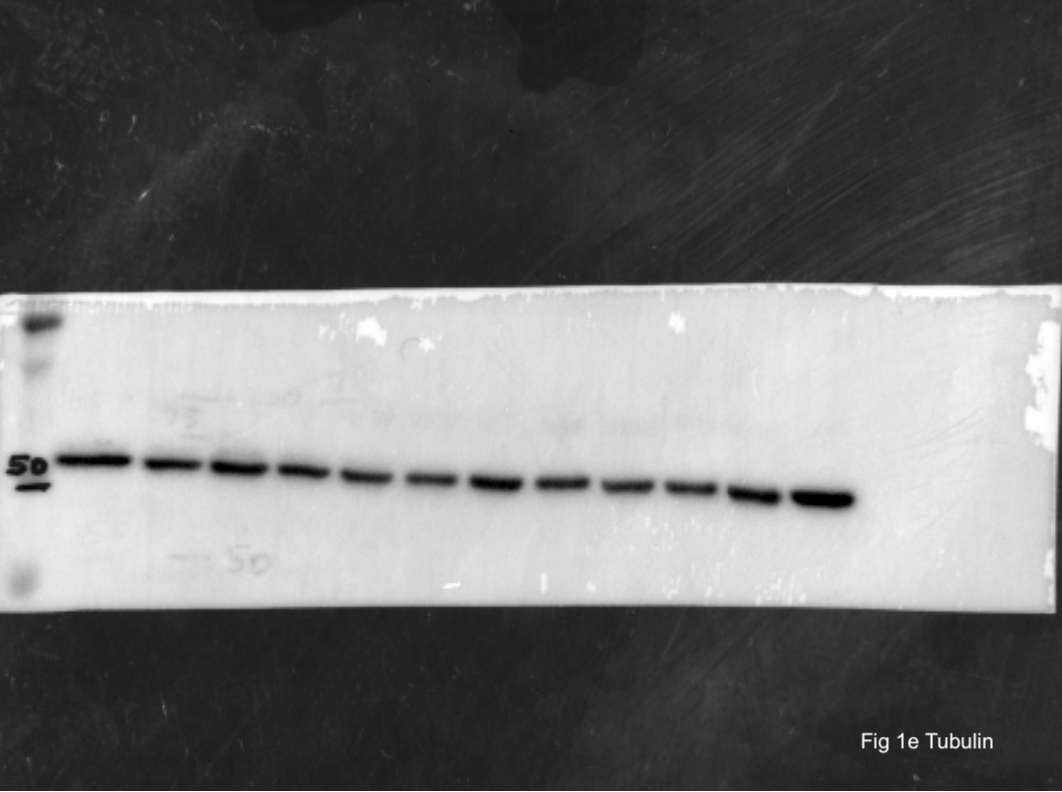


**Figure S8.** Whole blot image of Tubulin from Figure 1E.


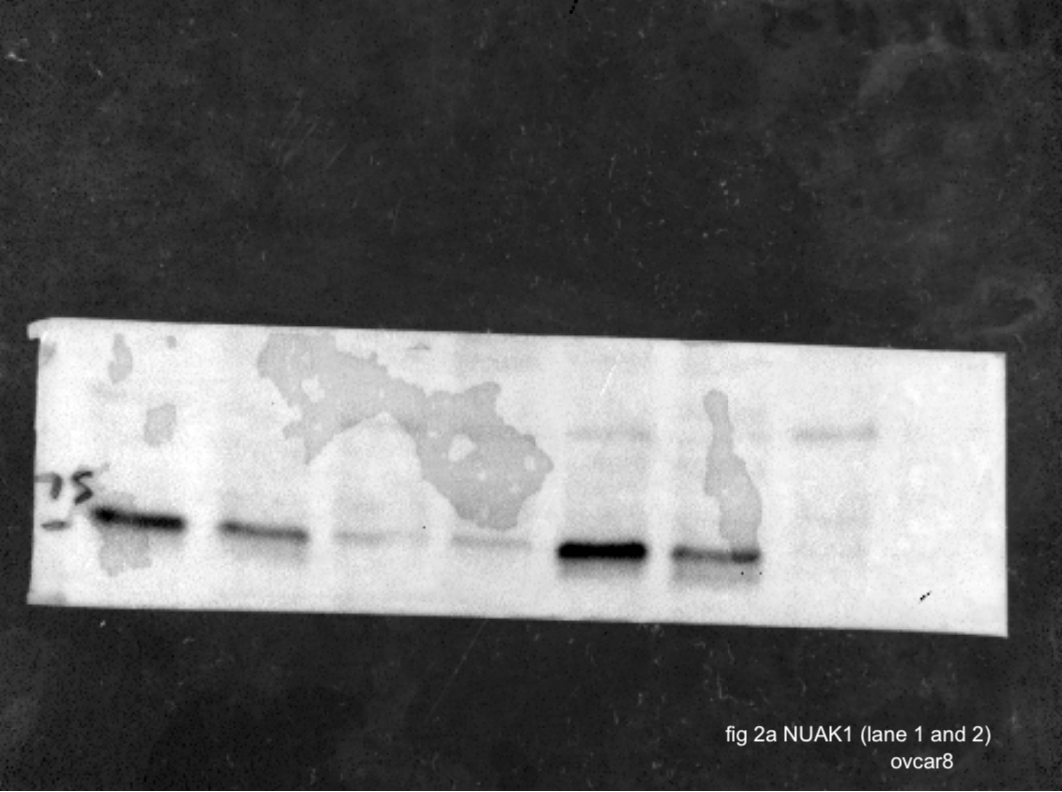


**Figure S9.** Whole blot image of NUAK1 OVCAR8 (lane 1 and 2) from Figure 2A.


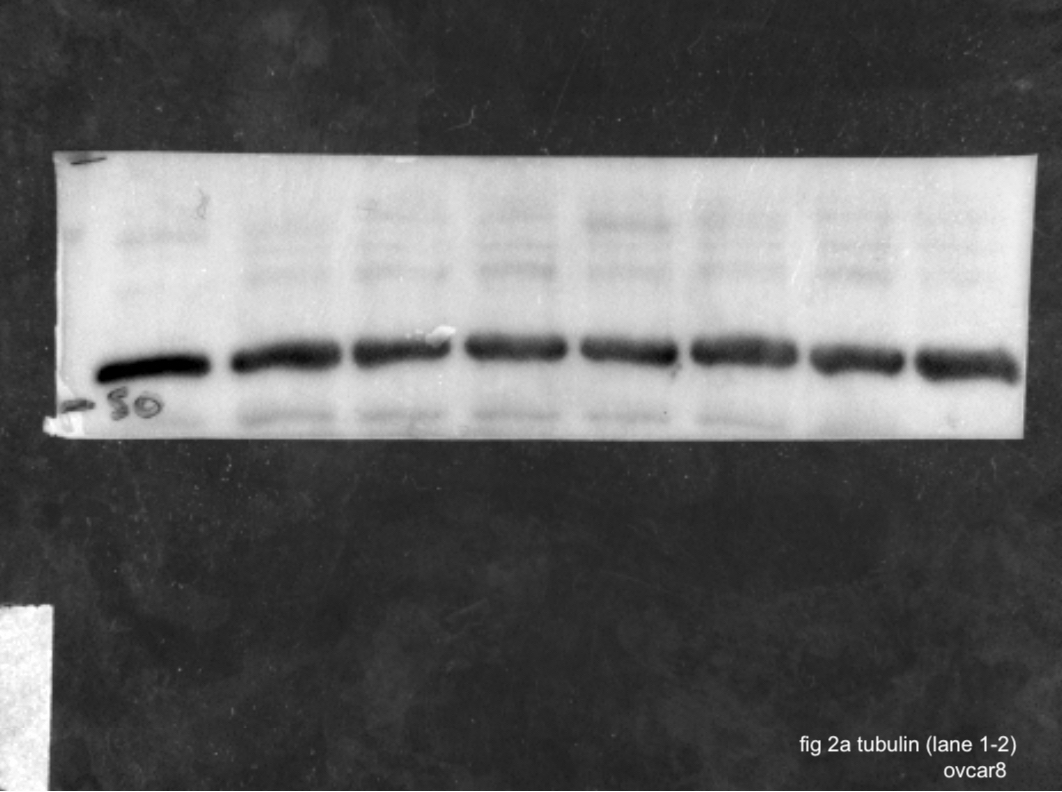


**Figure S10.** Whole blot image of Tubulin OVACR8 (lane 1 and 2) from Figure 2A.


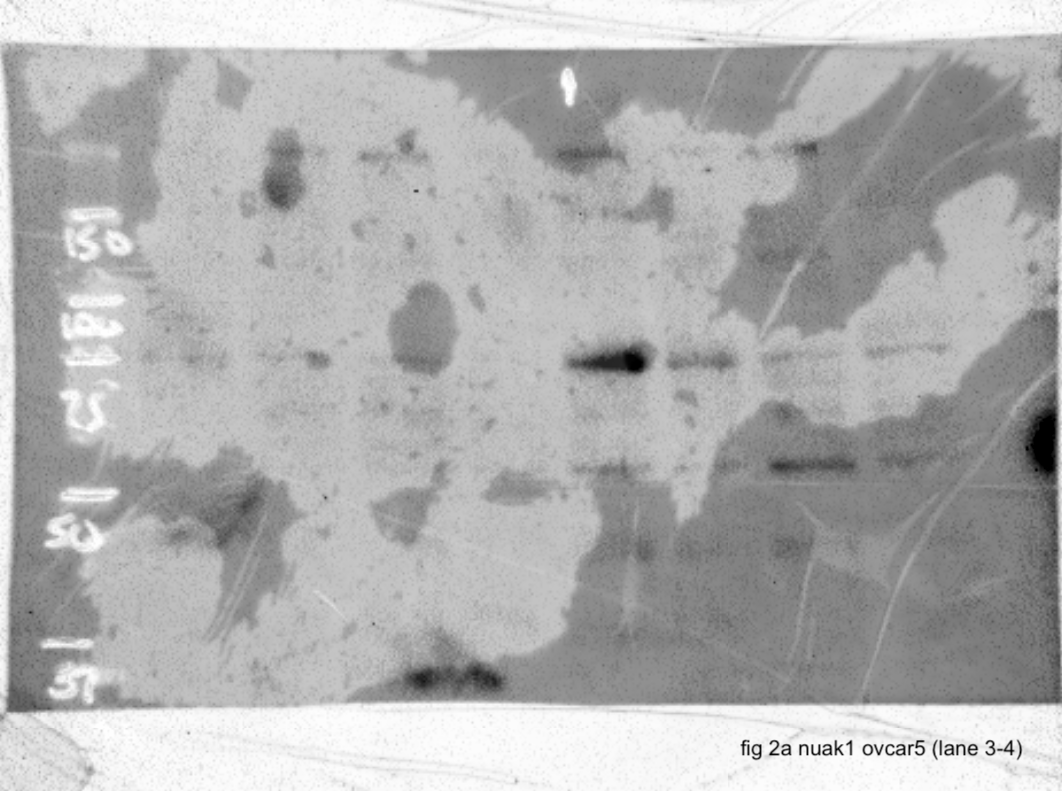


**Figure S11.** Whole blot image of NUAK1 OVCAR5 (lane 3 and 4) from Figure 2A.


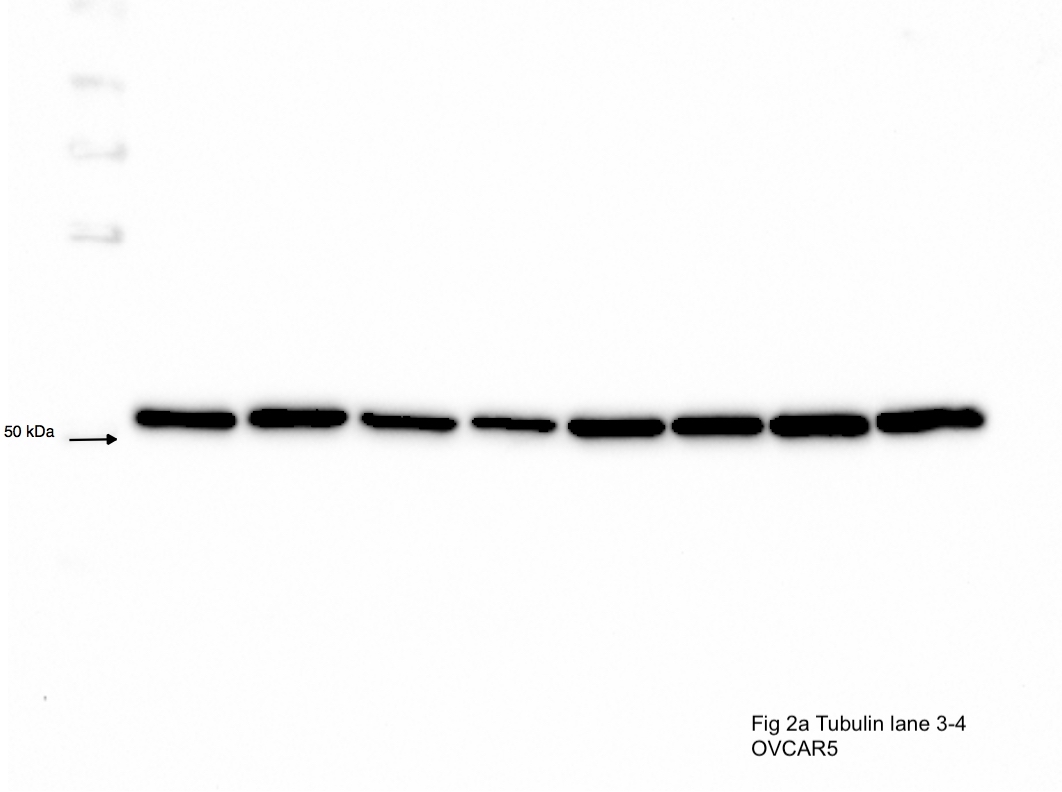


**Figure S12.** Whole blot image of Tubulin OVACR5 (lane 3 and 4) from Figure 2A.


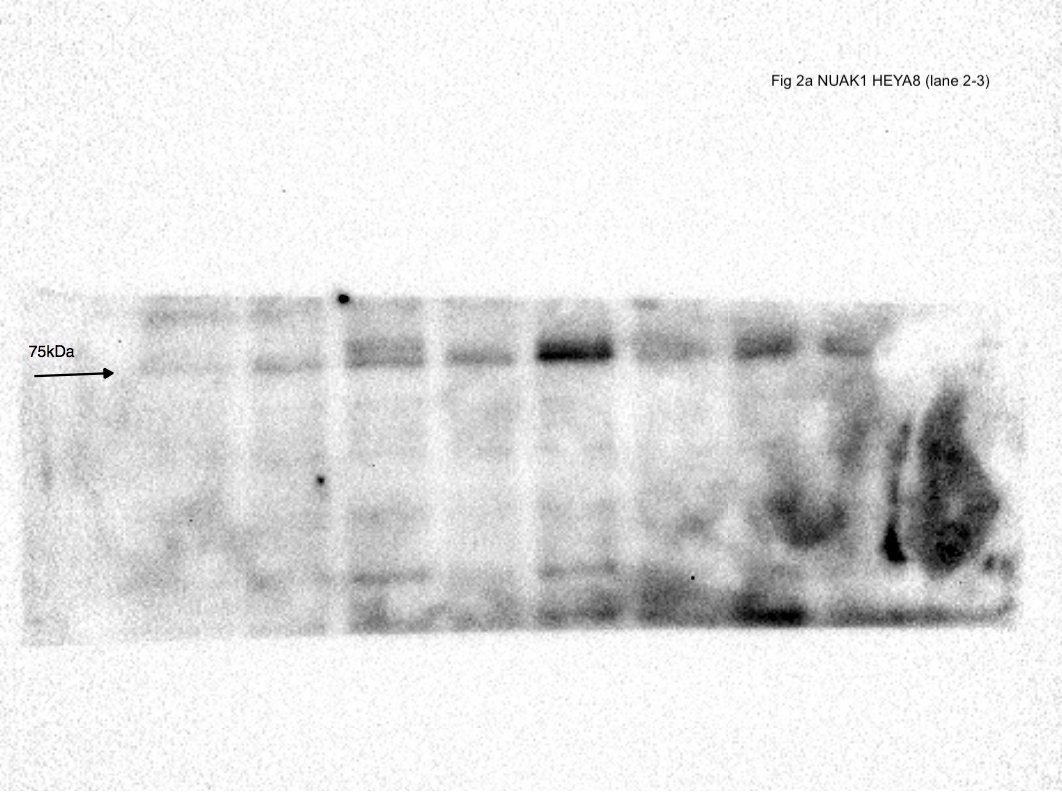


**Figure S13.** Whole blot image of NUAK1 HEYA8 (lane 2 and 3) from Figure 2A.


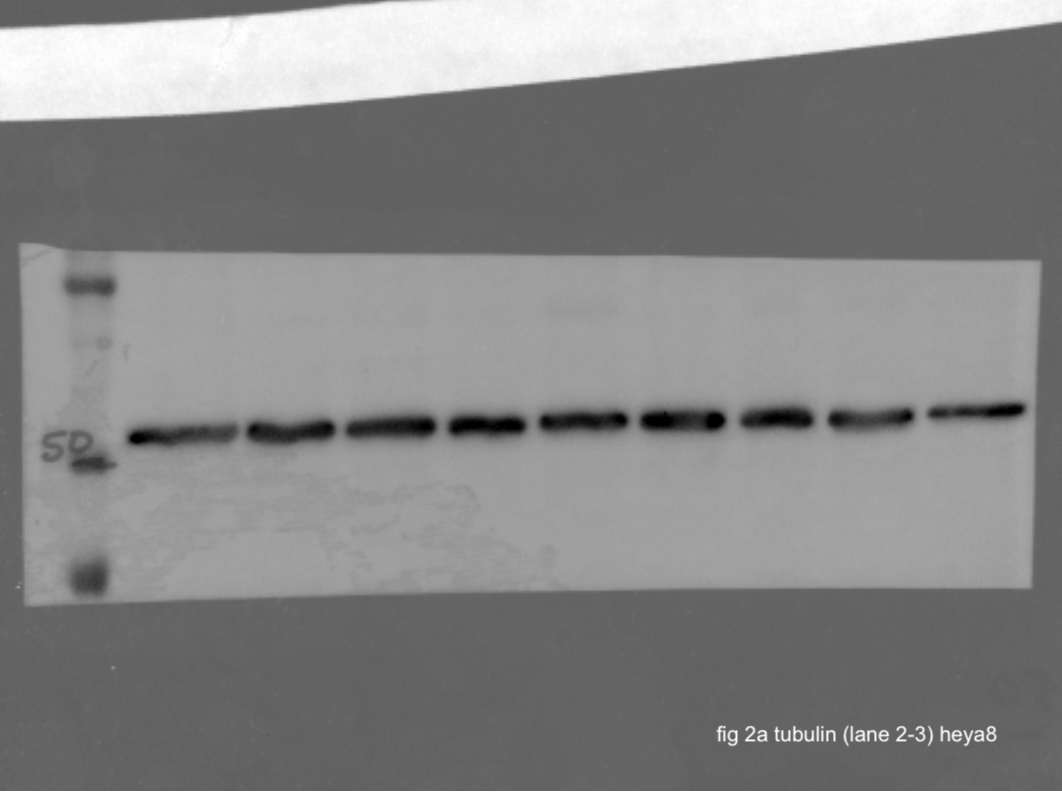


**Figure S14.** Whole blot image of Tubulin HEYA8 (lane 2 and 3) from Figure 2A.


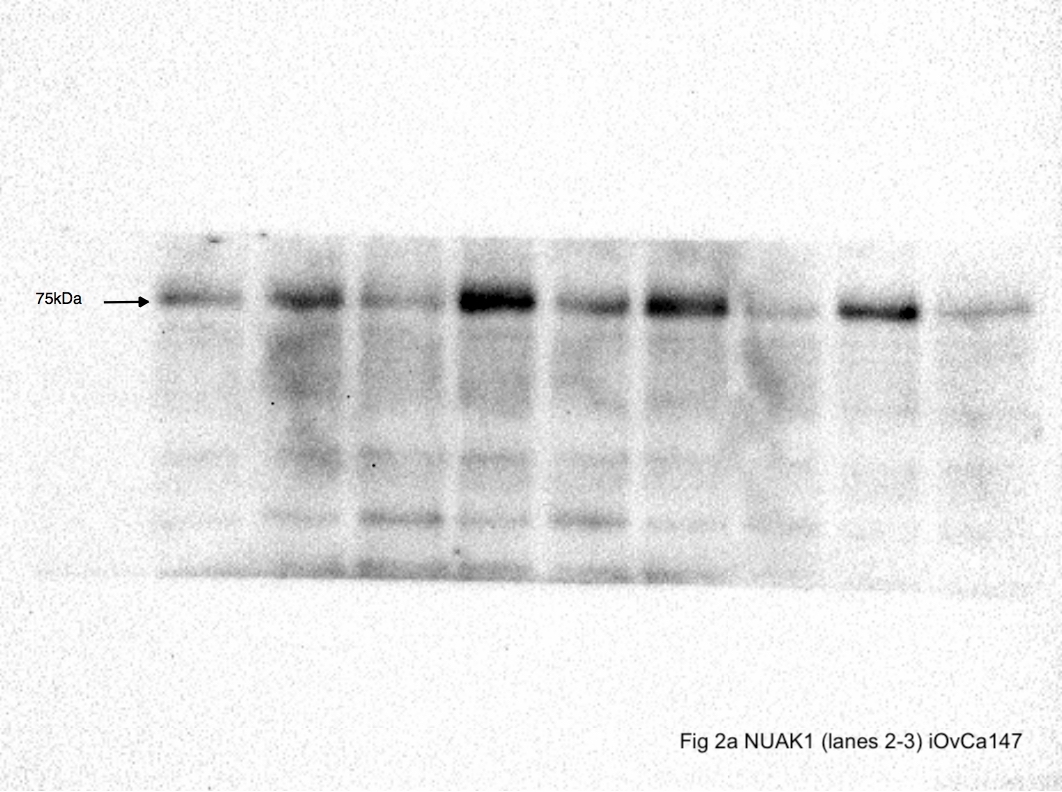


**Figure S15.** Whole blot image of NUAK1 iOvCa147 (lane 2 and 3) from Figure 2A.


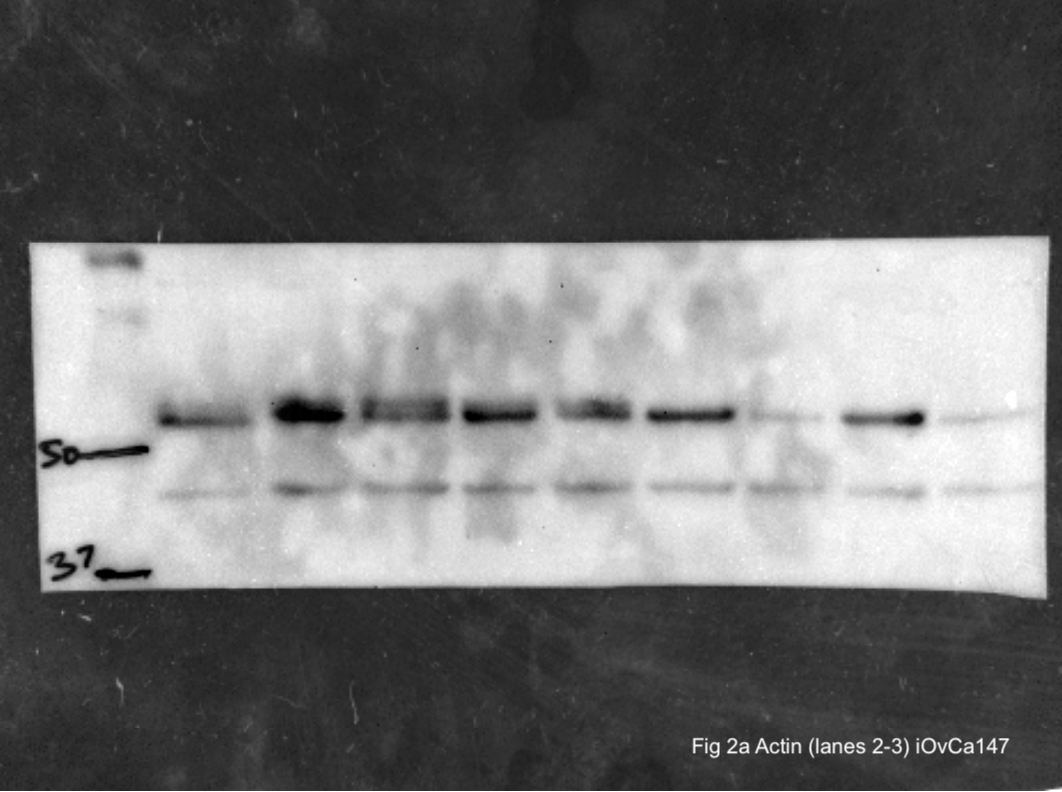


**Figure S16.** Whole blot image of Actin IOvCa147 (lane 2 and 3) from Figure 2A.


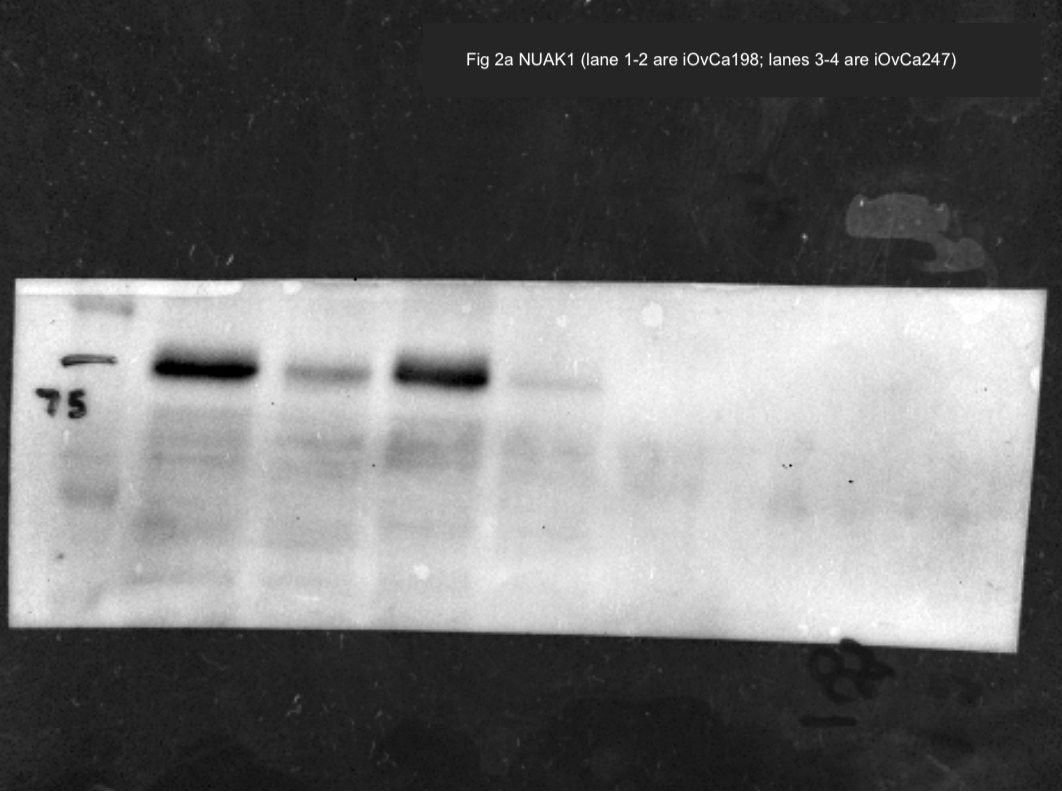


**Figure S17.** Whole blot image of NUAK1 (lanes 1, 2 are in iOvCa198 and lanes 3, 4 are in iOvCa247) from Figure 2A.


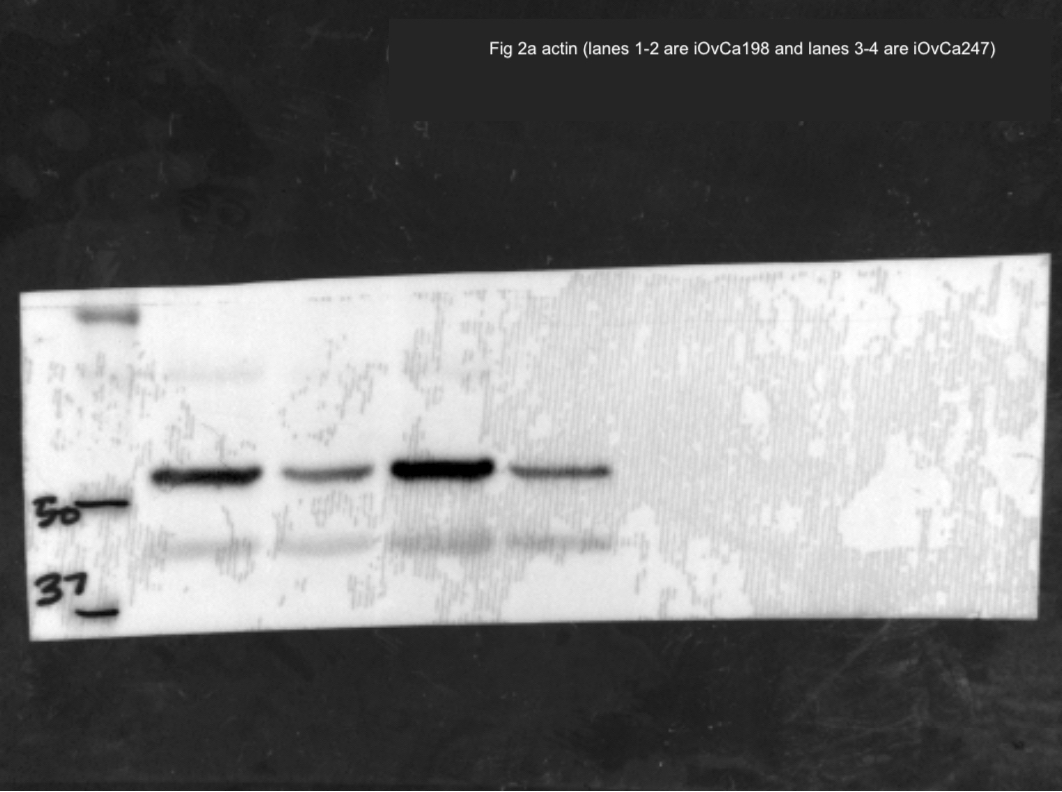


**Figure S18.** Whole blot image of Actin (lanes (lanes 1, 2 are in iOvCa198 and lanes 3, 4 are in iOvCa247) from Figure 2A.


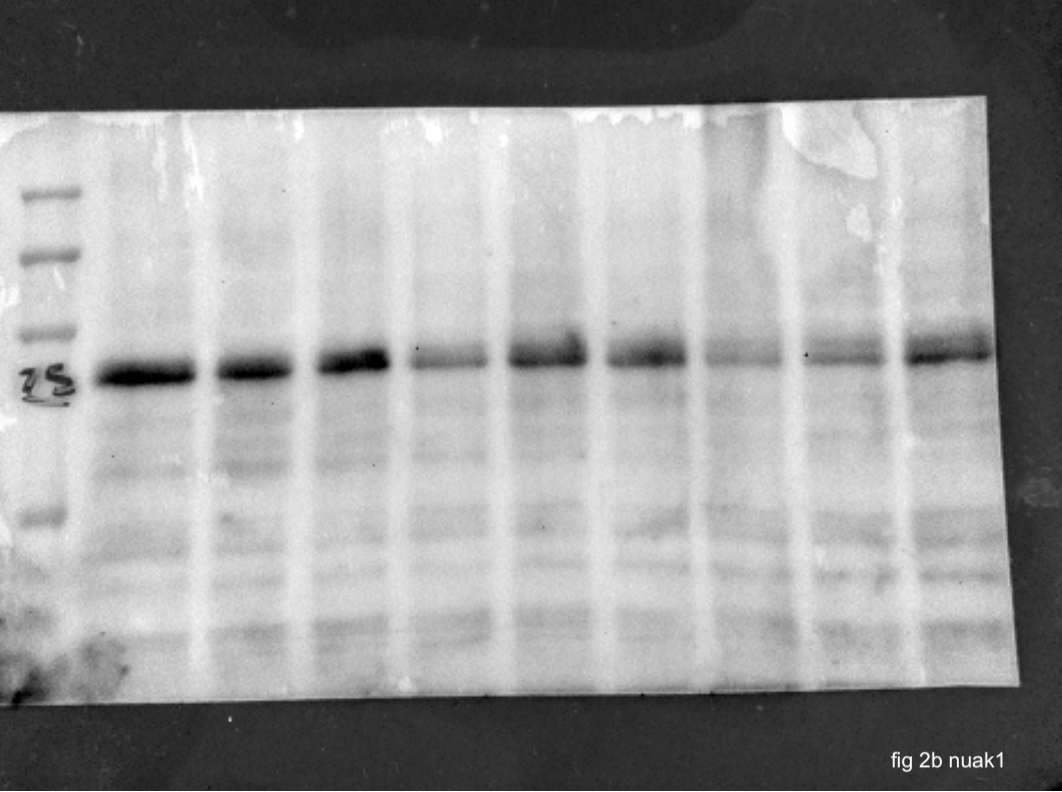


**Figure S19.** Whole blot image of NUAK1 from Figure 2B.


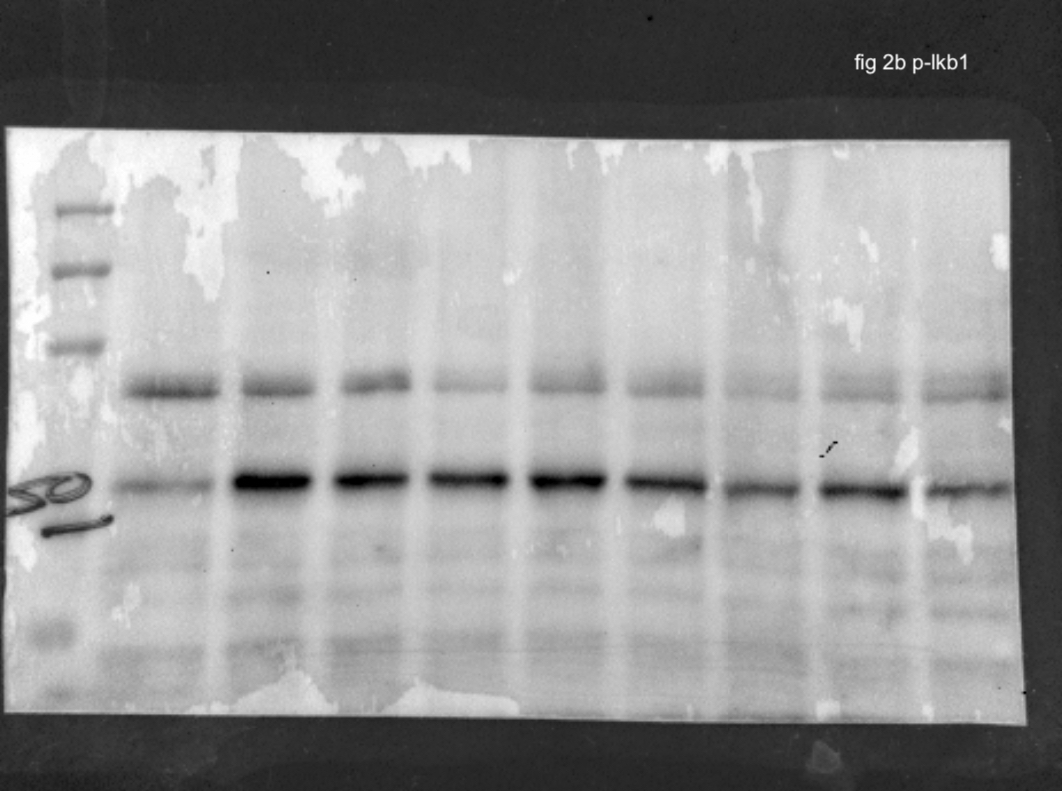


**Figure S20.** Whole blot image of p-LKB1 from Figure 2B.


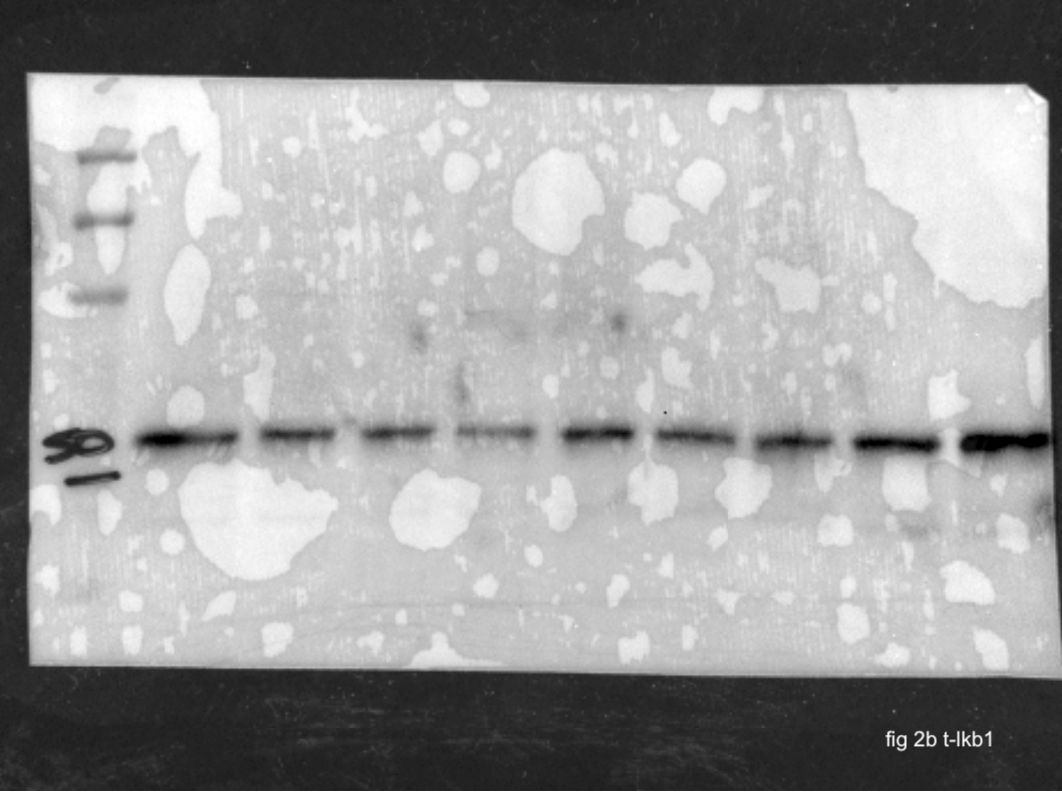


**Figure S21.** Whole blot image of t-LKB1 from Figure 2B.


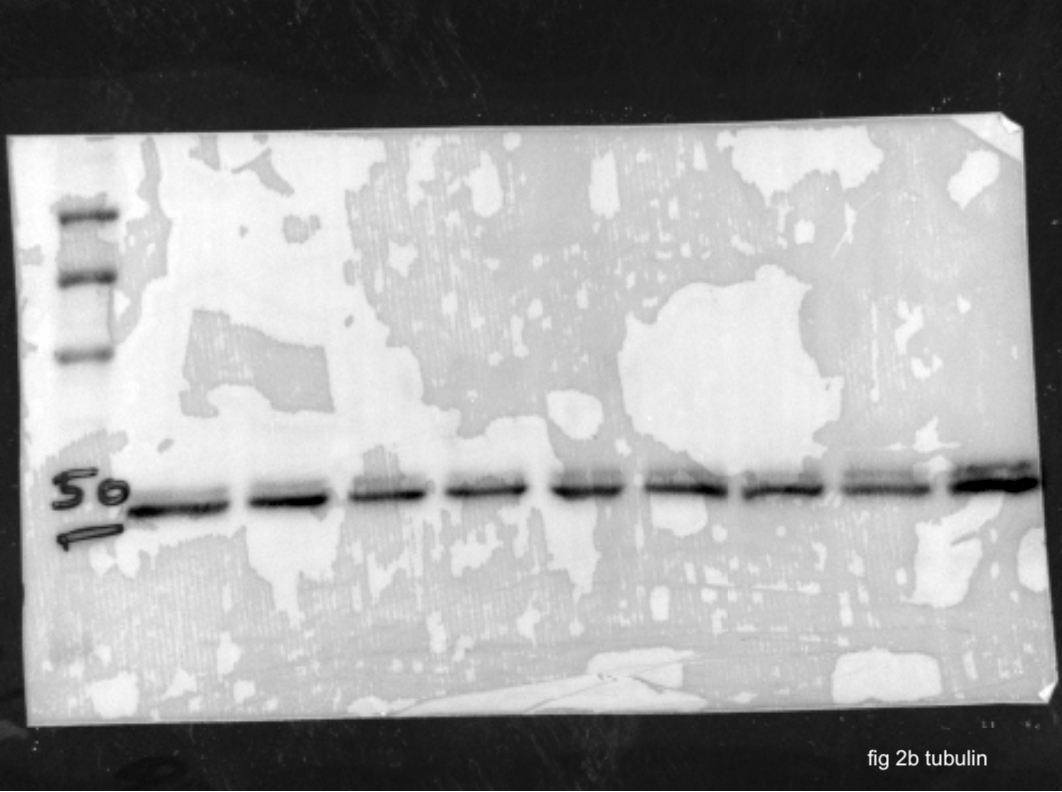


**Figure S22.** Whole blot image of Tubulin from Figure 2B.


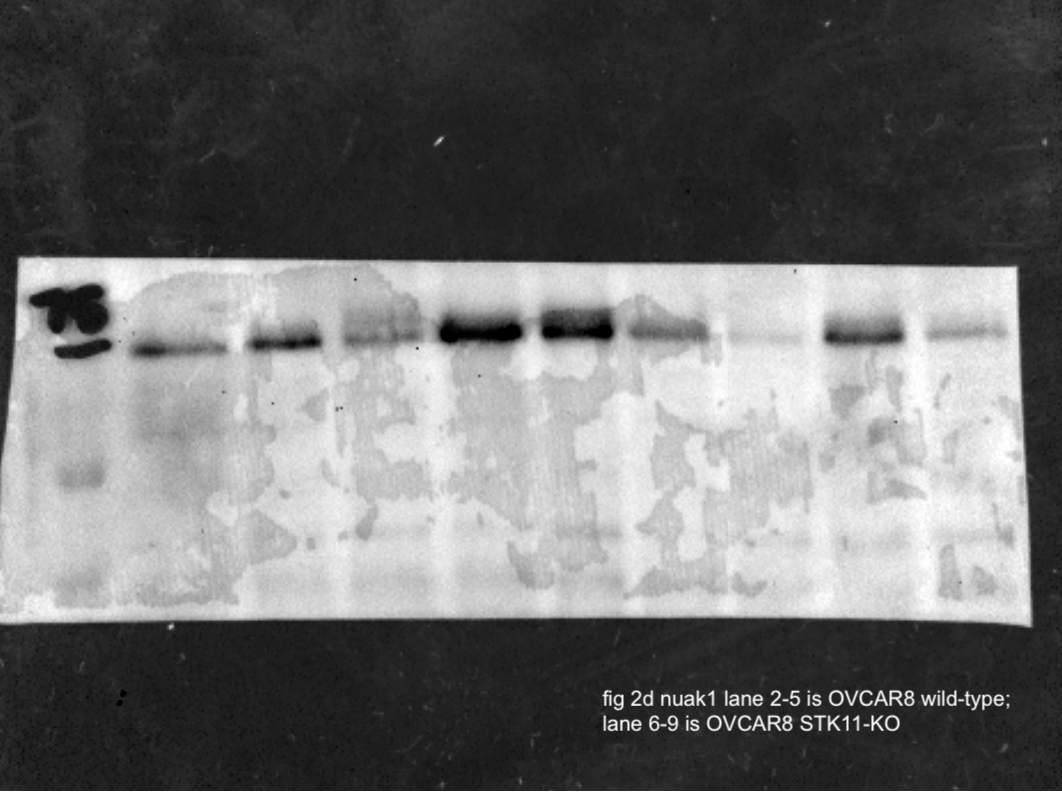


**Figure S23.** Whole blot image of NUAK1 (lanes 2–5 are OVCAR8 wild-type and lanes 6–9 are OVCAR8 STK11-KO) from Figure 2D.


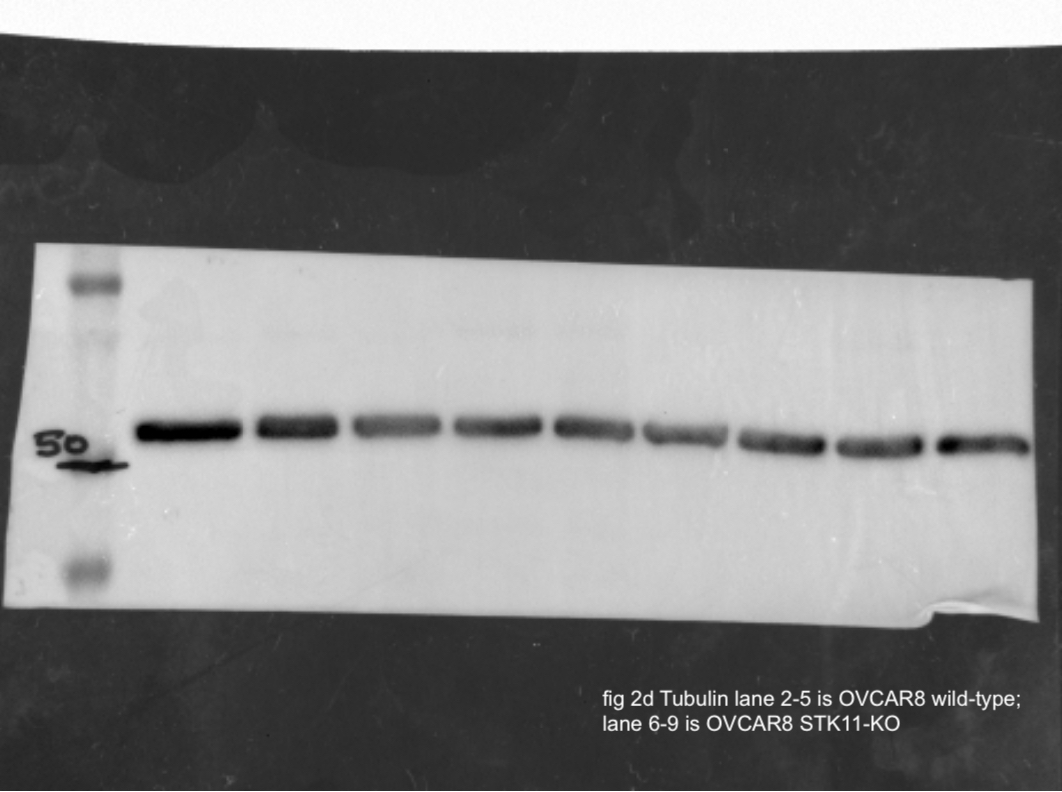


**Figure S24.** Whole blot image of Tubulin (lanes 2–5 are OVCAR8 wild-type and lanes 6–9 are OVCAR8 STK11-KO) from Figure 2D.


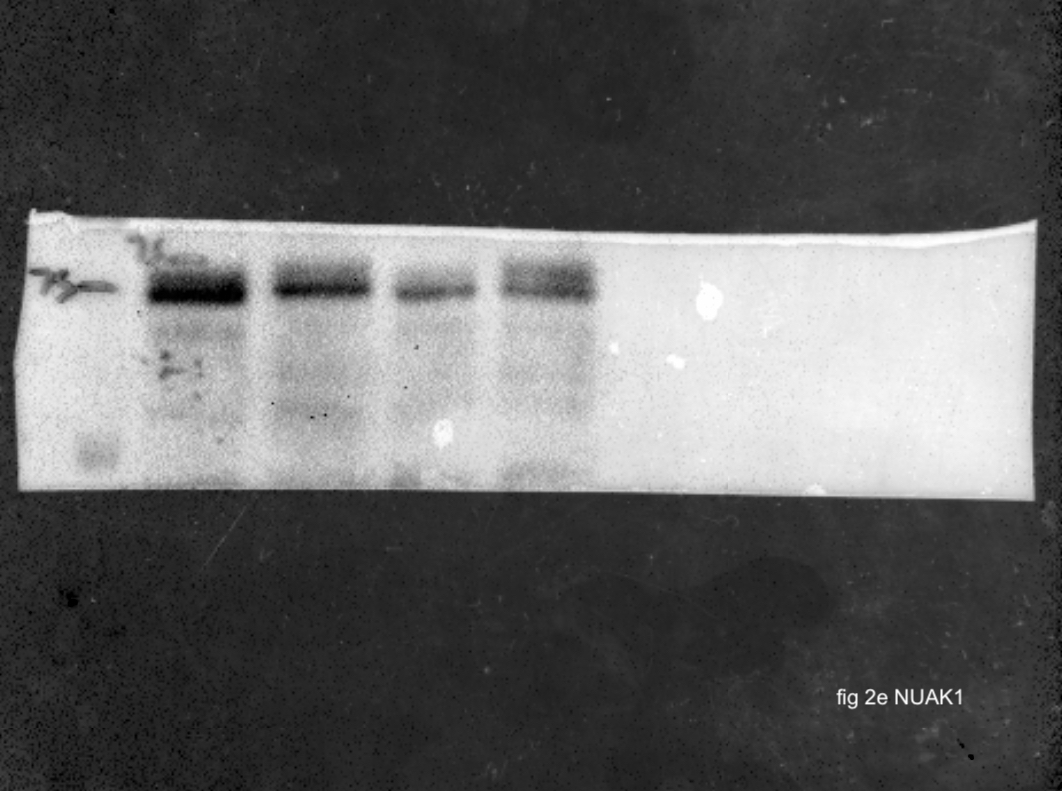


**Figure S25.** Whole blot image of NUAK1 from Figure 2E.


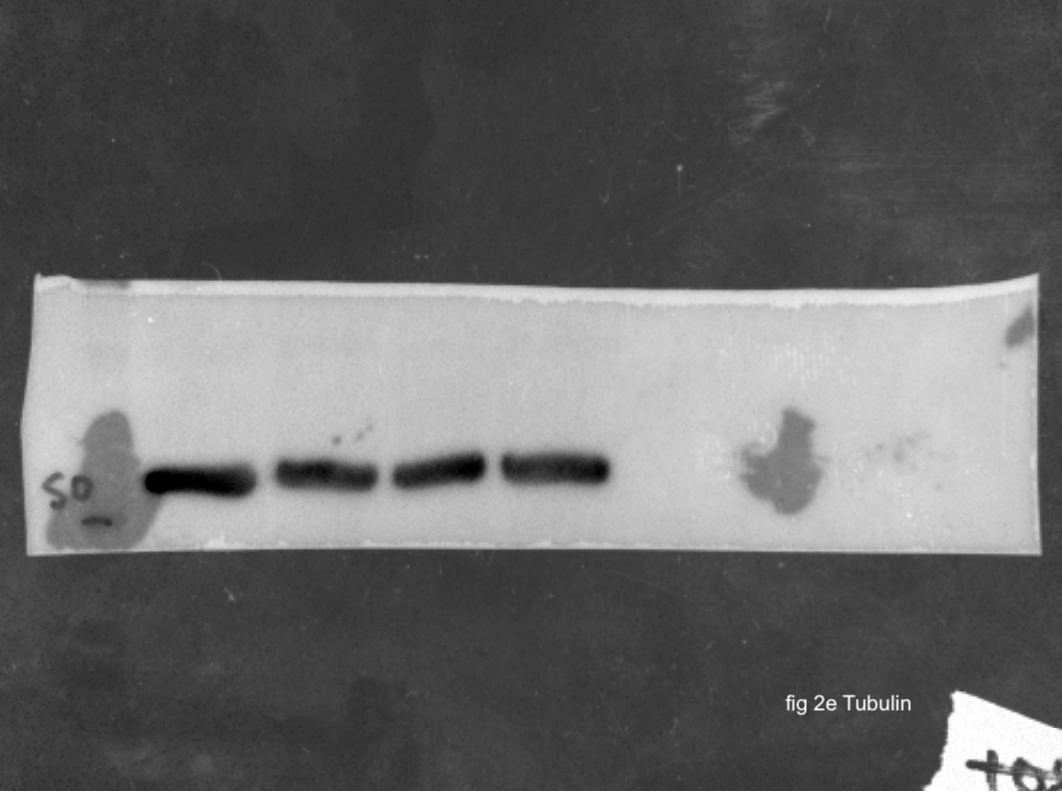


**Figure S26.** Whole blot image of Tubulin from Figure 2E.


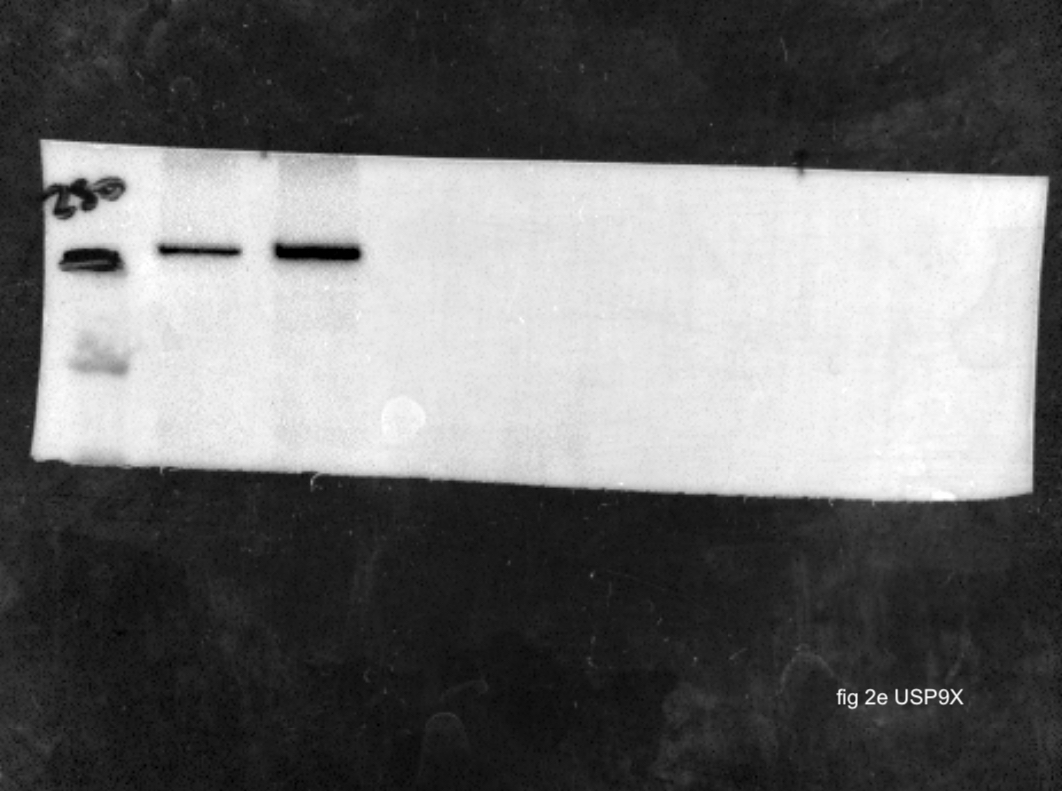


**Figure S27.** Whole blot image of USP9X from Figure 2E.


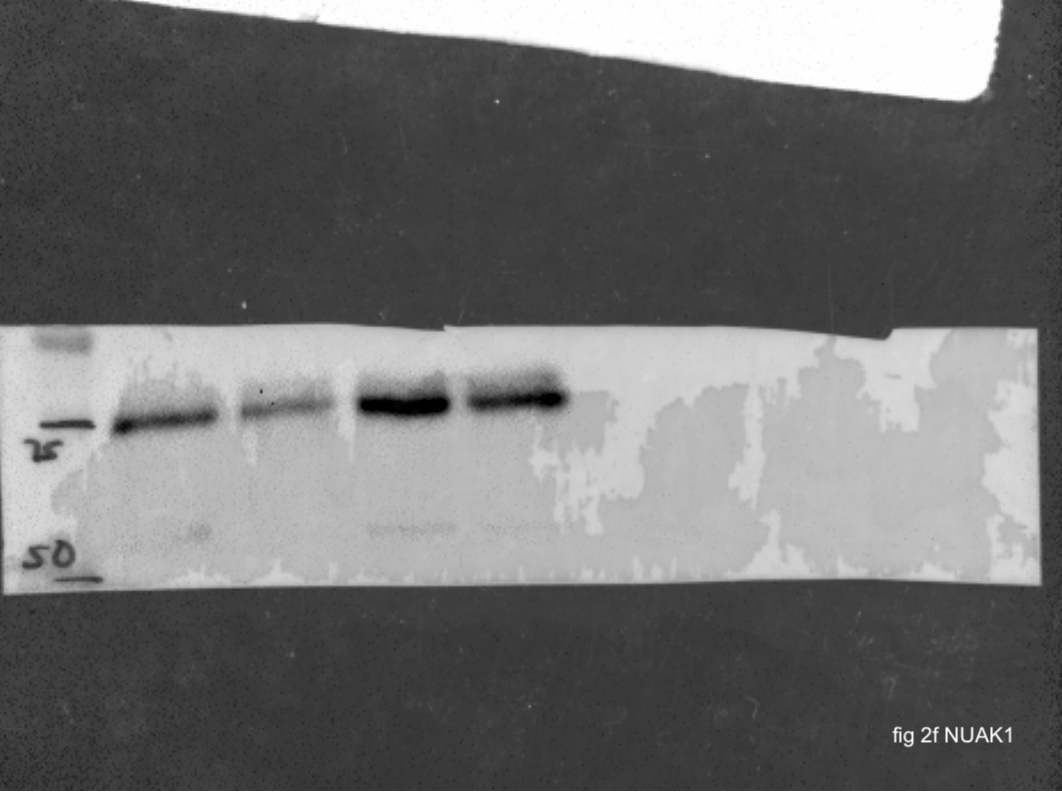


**Figure S28.** Whole blot image of NUAK1 from Figure 2F.


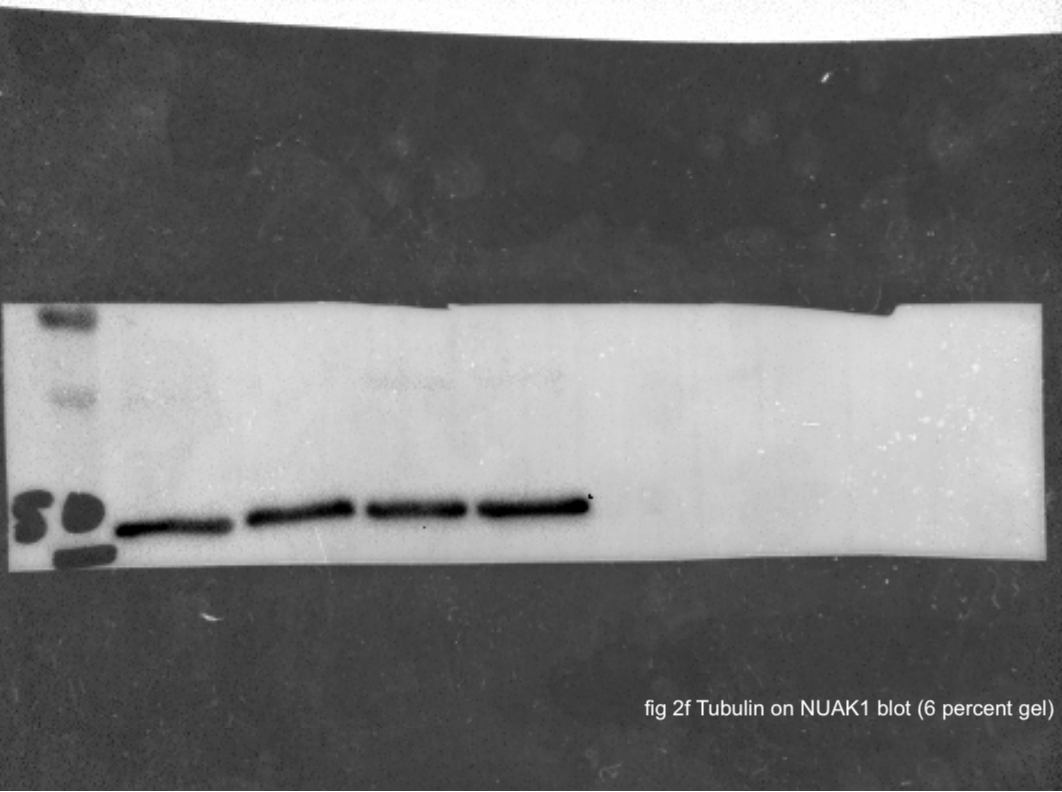


**Figure S29.** Whole blot image of Tubulin on NUAK1 blot (6% gel) from Figure 2F.


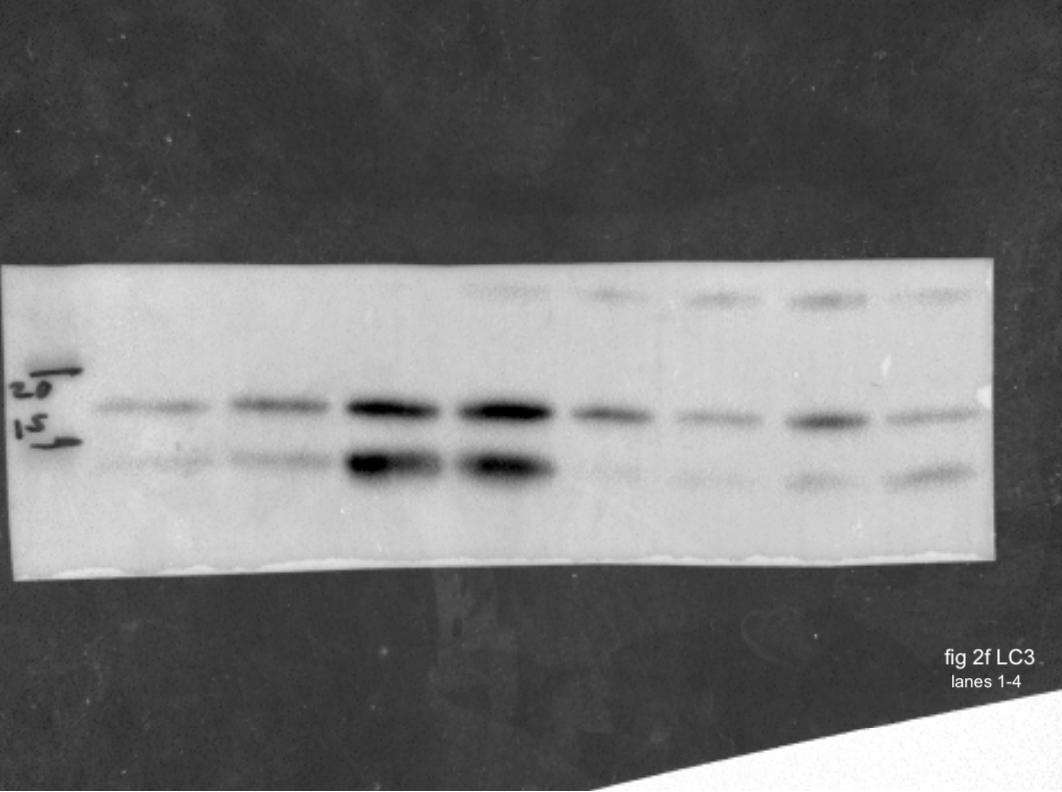


**Figure S30.** Whole blot image of LC3 (lanes 1–4) form Figure 2F.


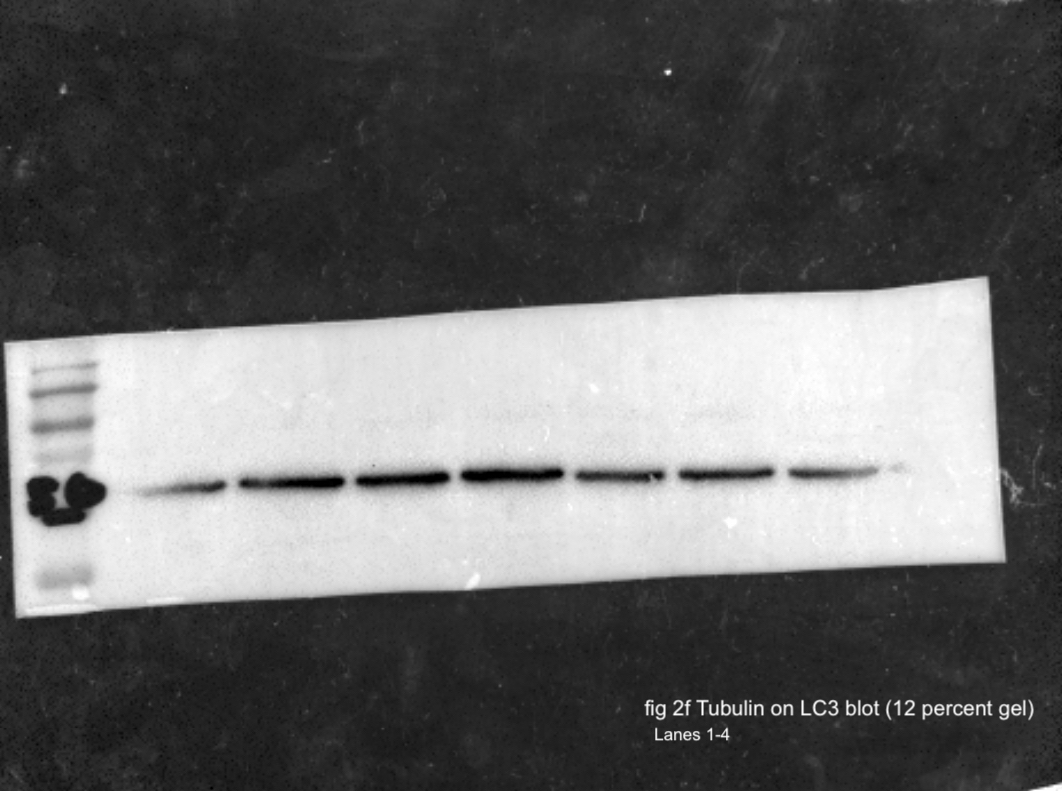


**Figure S31.** Whole blot image of Tubulin on LC3 blot (12% gel, lanes 1–4) from Figure 2F.


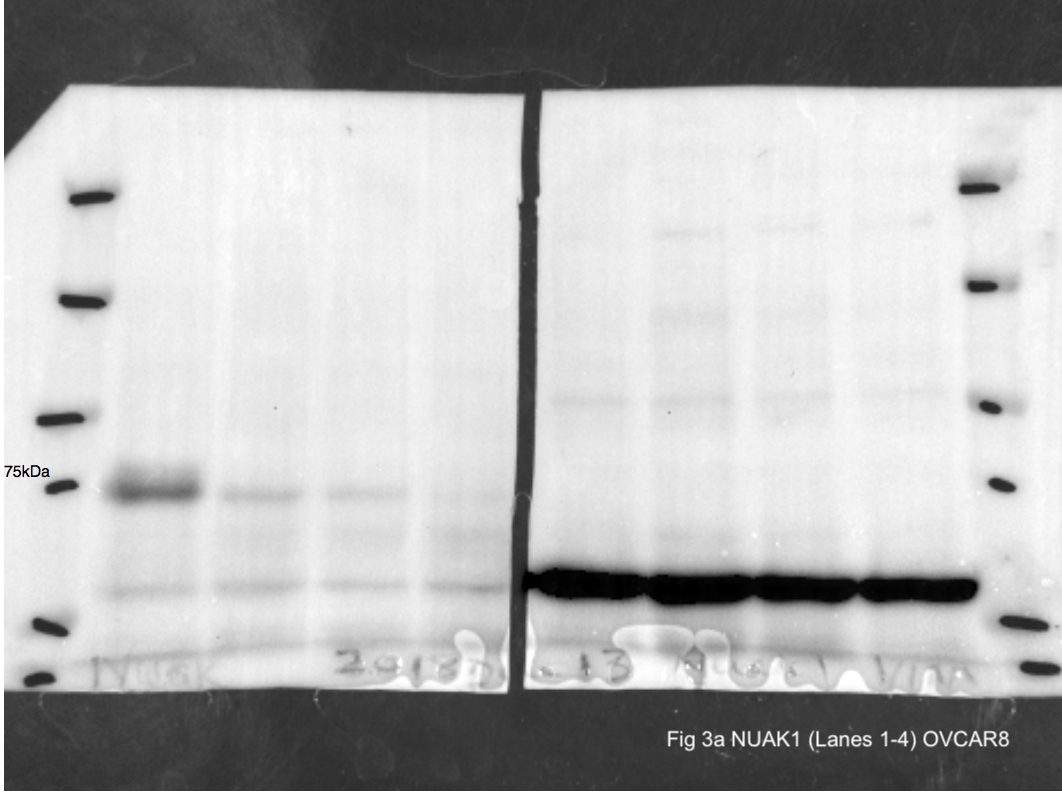


**Figure S32.** Whole blot image of NUAK1 OVCAR8 (lanes 1–4) form Figure 3A.


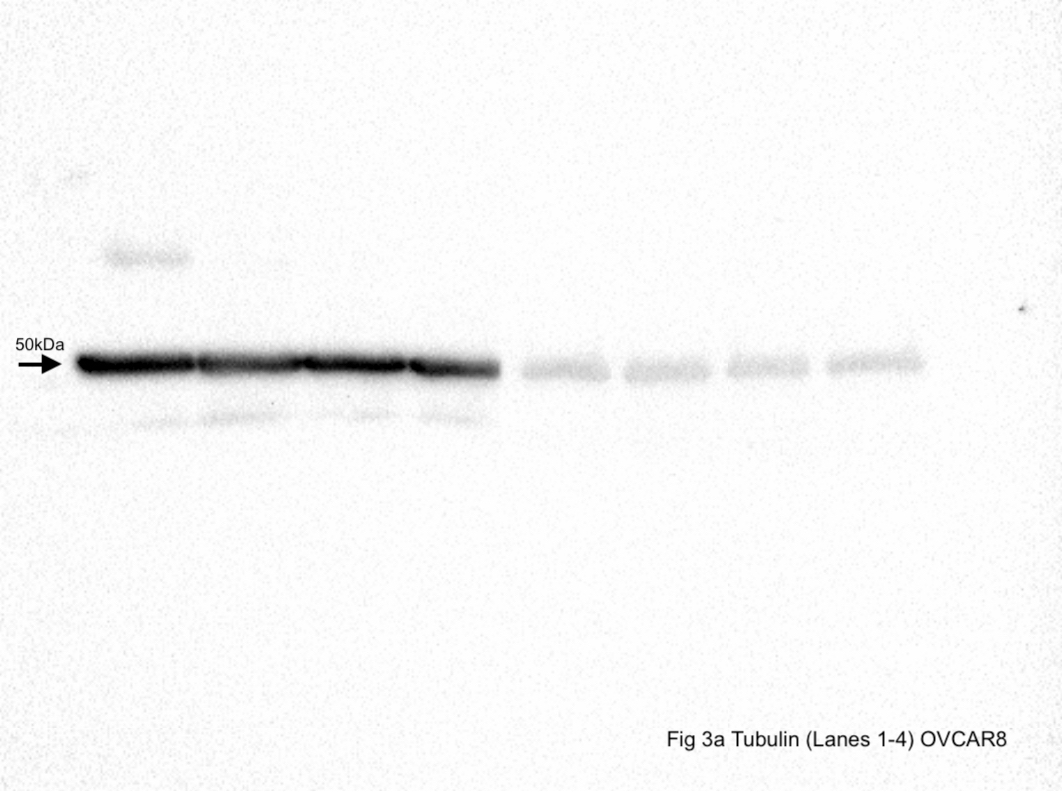


**Figure S33.** Whole blot image of Tubulin OVCAR8 (lanes 1–4) from Figure 3A.


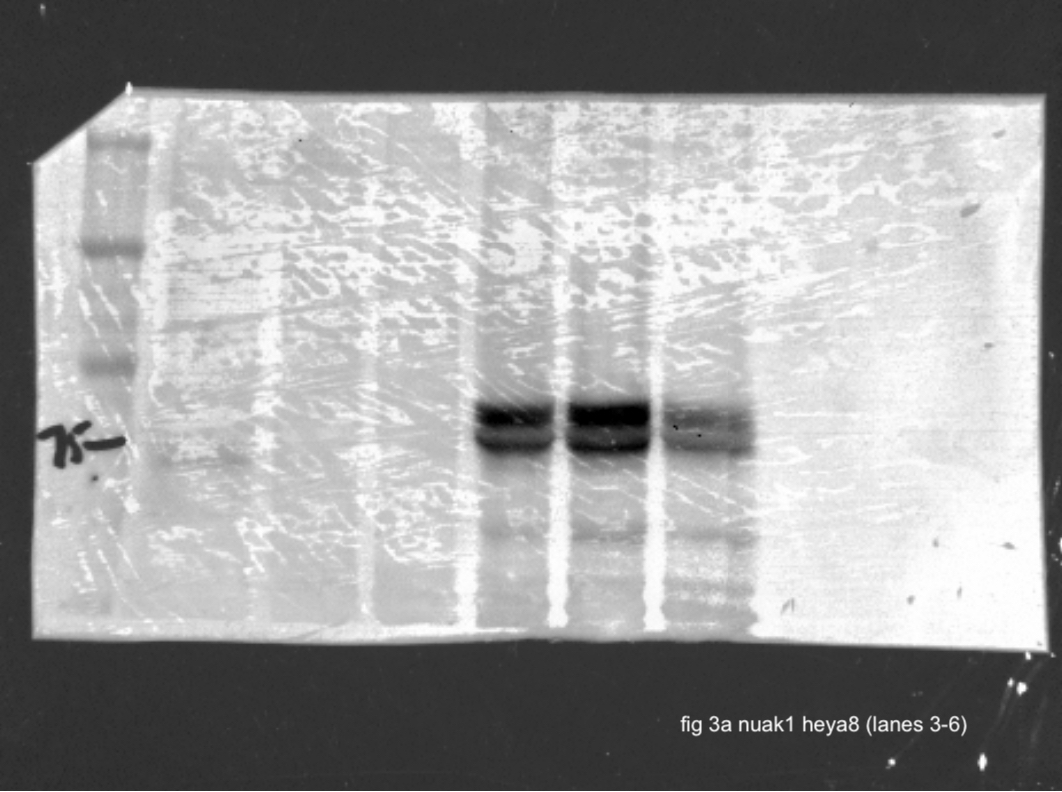


**Figure S34.** Whole blot image of NUAK1 HEYA8 (lanes 3–6) from Figure 3A.


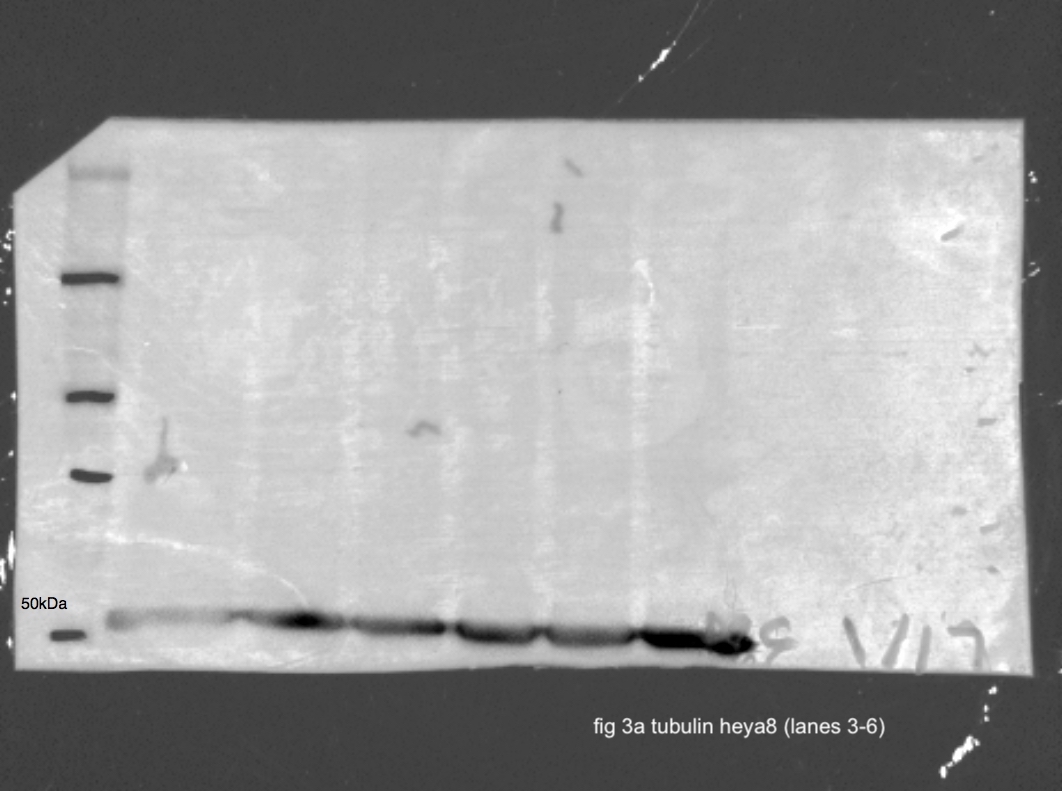


**Figure S35.** Whole blot image of Tubulin HEYA8 (lanes 3–6) form Figure 3A.


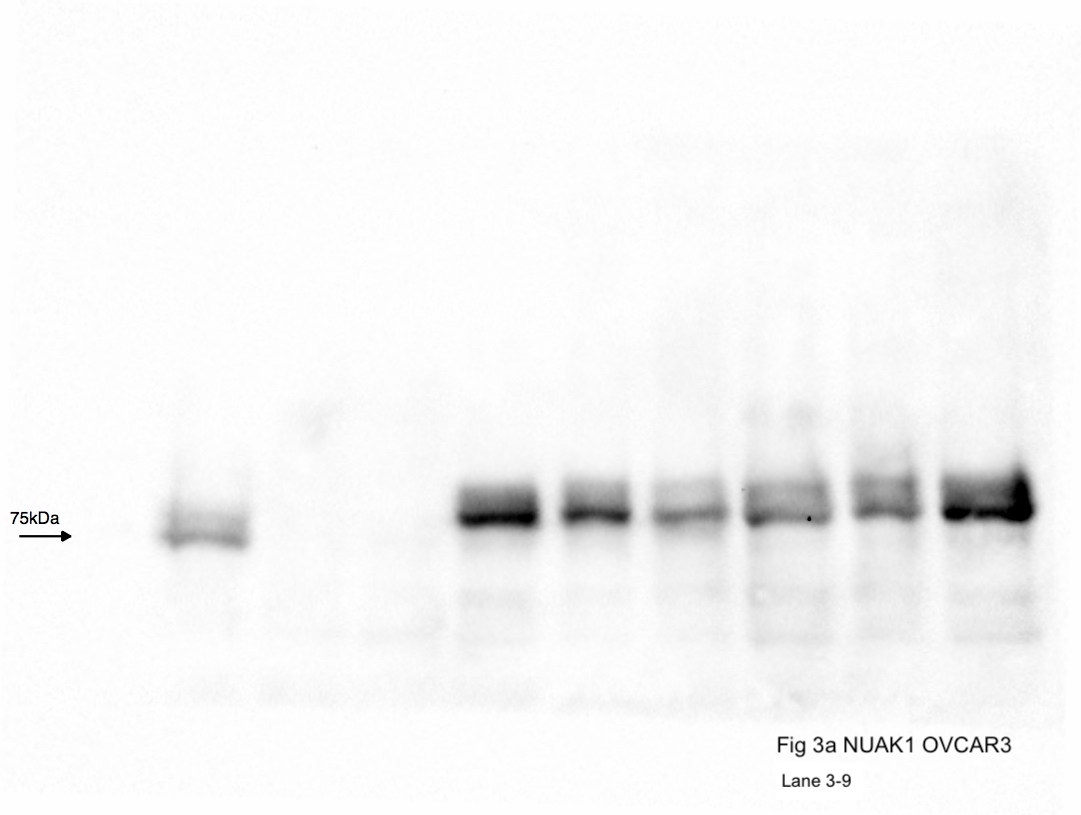


**Figure S36.** Whole blot image of NUAK1 OVCAR3 (lane 3–9) from Figure 3A.


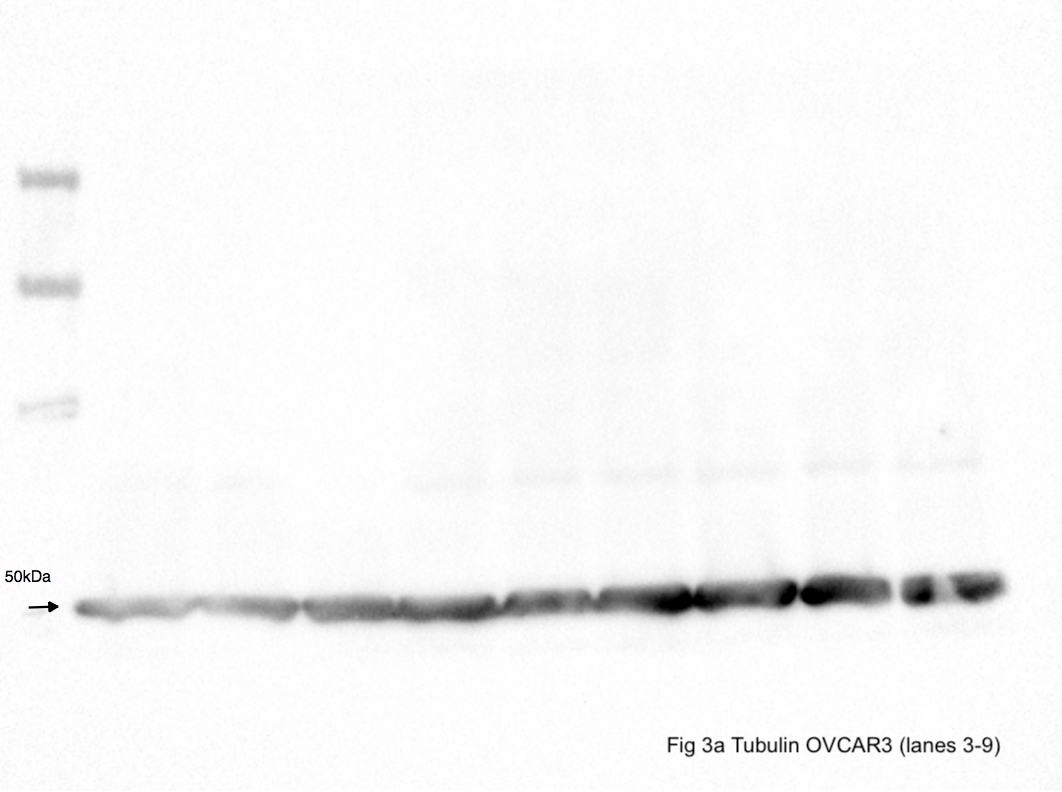


**Figure S37.** Whole blot image of Tubulin OVCAR3 (lanes 3–9) from Figure 3A.


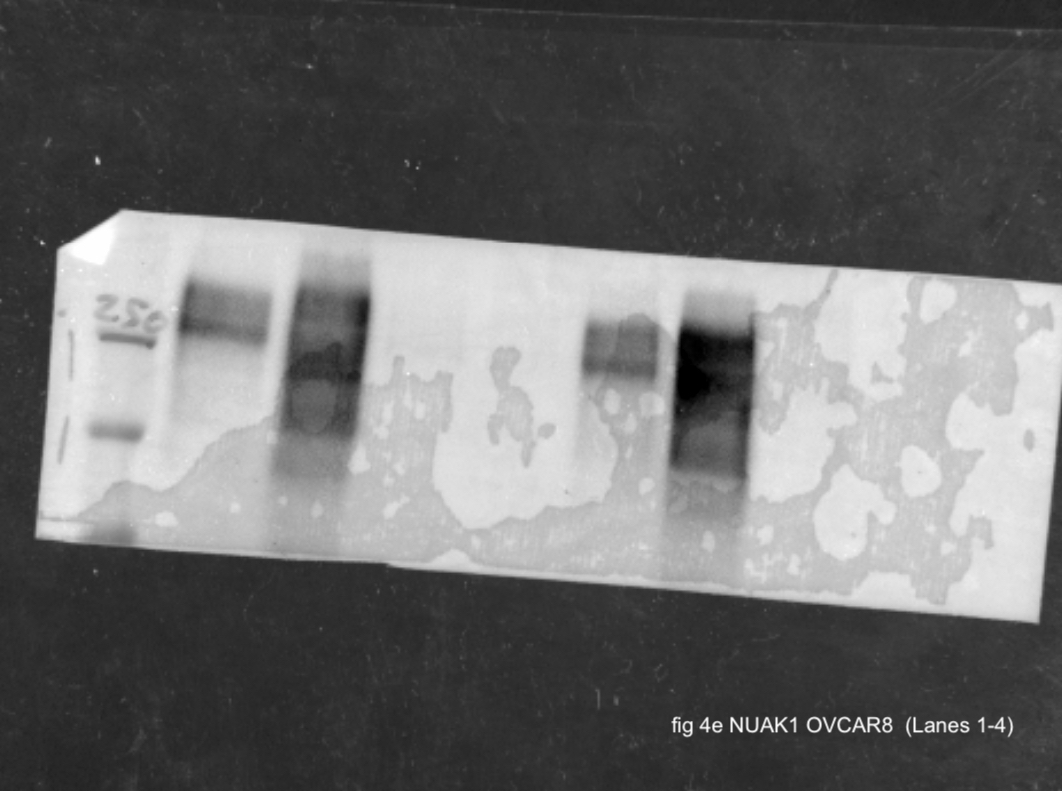


**Figure S38.** Whole blot image of NUAK1 OVCAR8 (lanes 1–4) form Figure 4E.


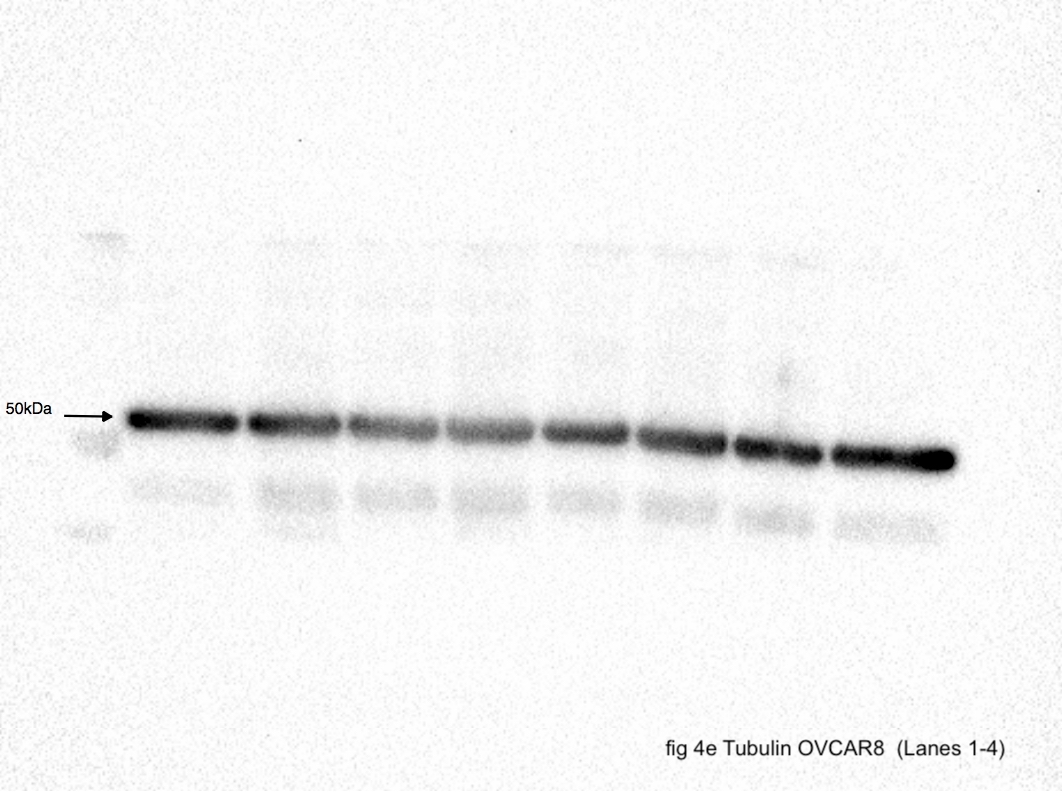


**Figure S39.** Whole blot image of Tubulin OVCAR8 (lanes 1–4) from Figure 4E.


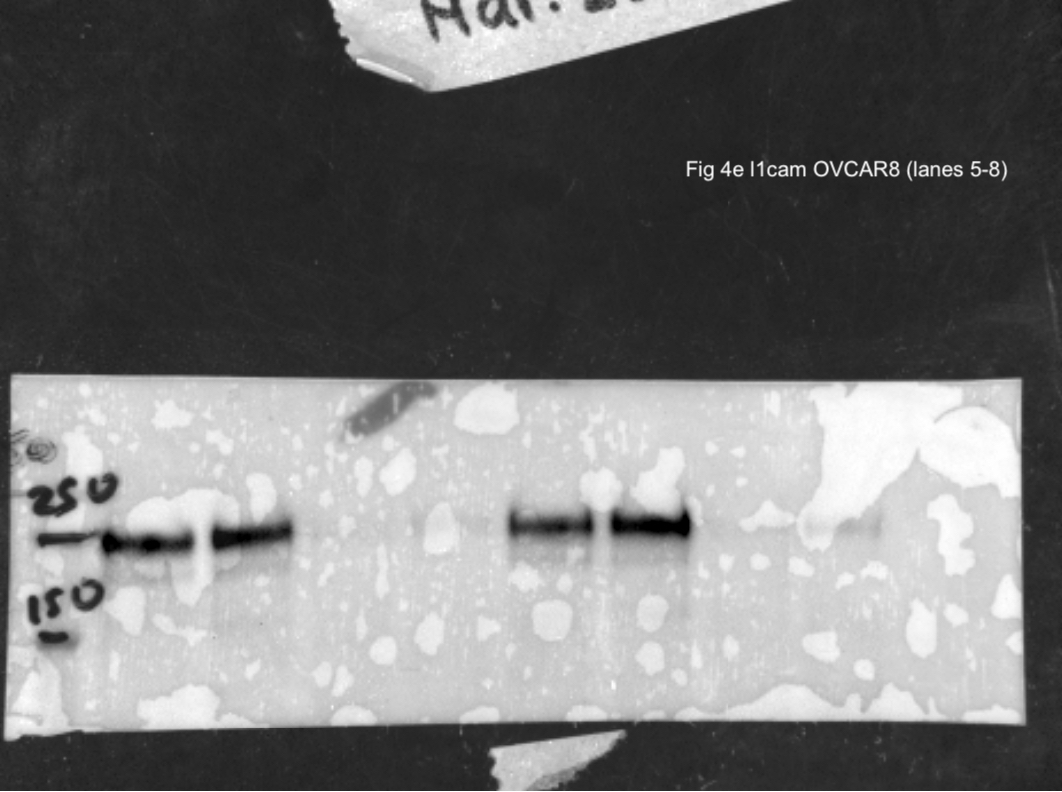


**Figure S40.** Whole blot image of L1CAM (lanes 5–8) from Figure 4E.


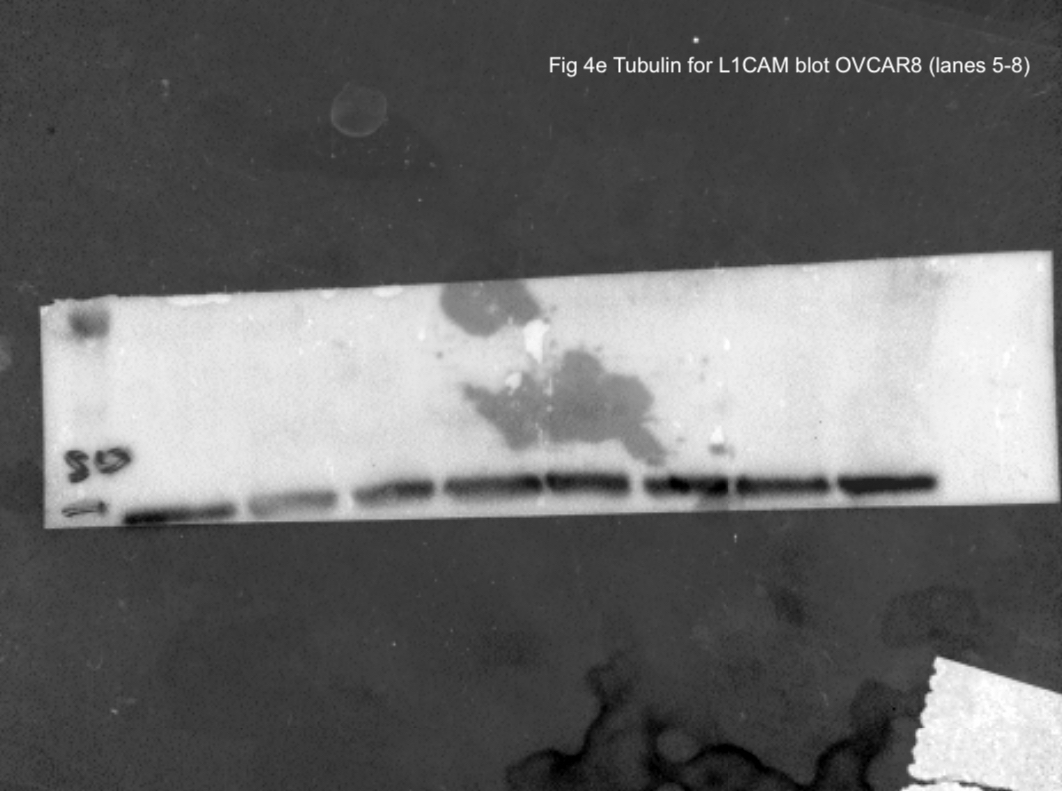


**Figure S41.** Whole blot image of Tubulin for L1CAM blot OVCAR8 (lanes 5–8) from Figure 4E.


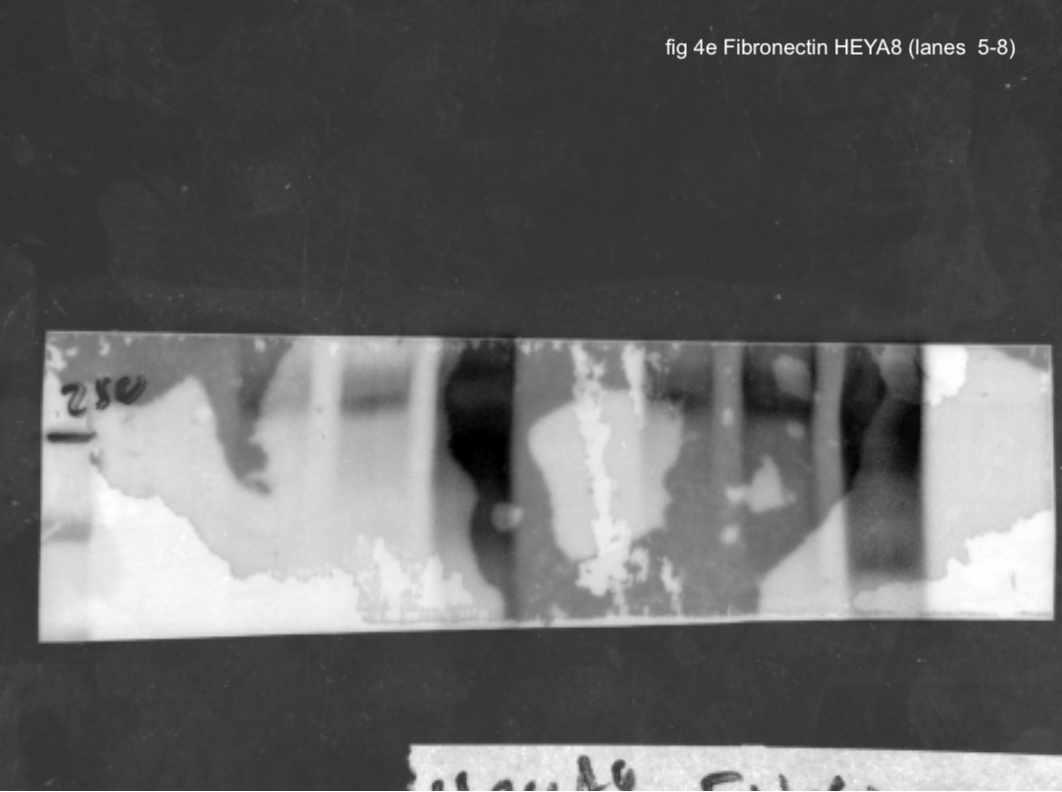


**Figure S42.** Whole blot image of Fibronectin HEYA8 (lanes 5–8) from Figure 4E.


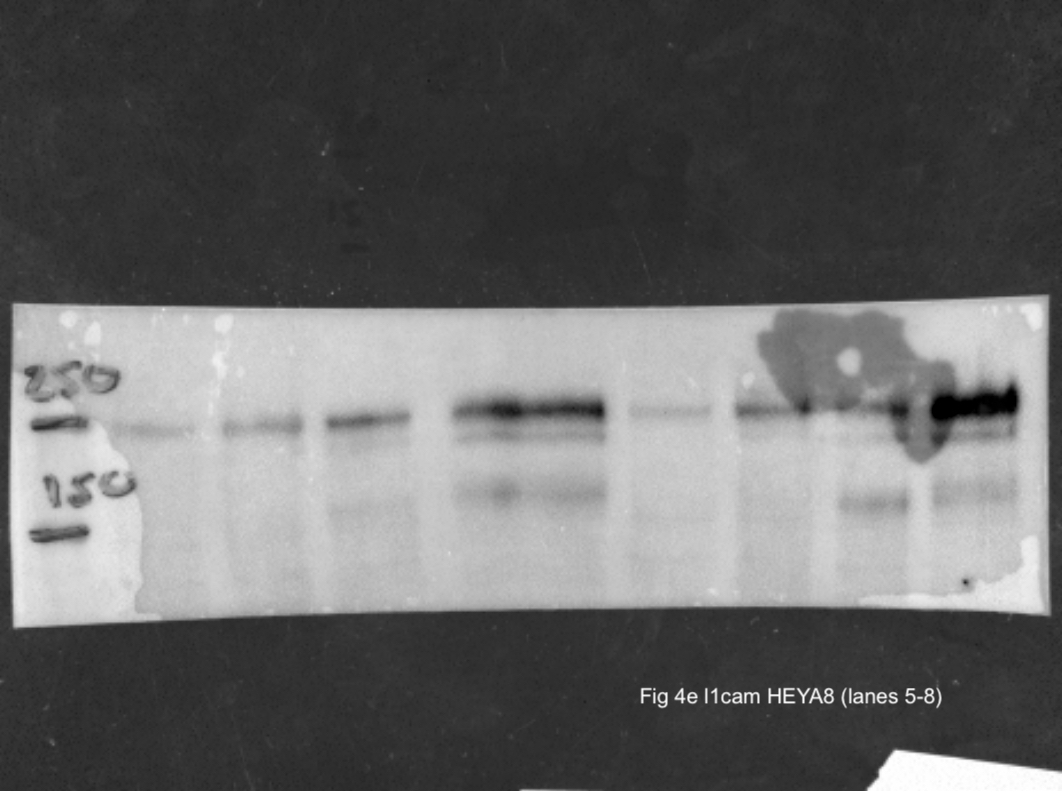


**Figure S43.** Whole blot image of L1CAM HEYA8 (lanes 5–8) from Figure 4E.


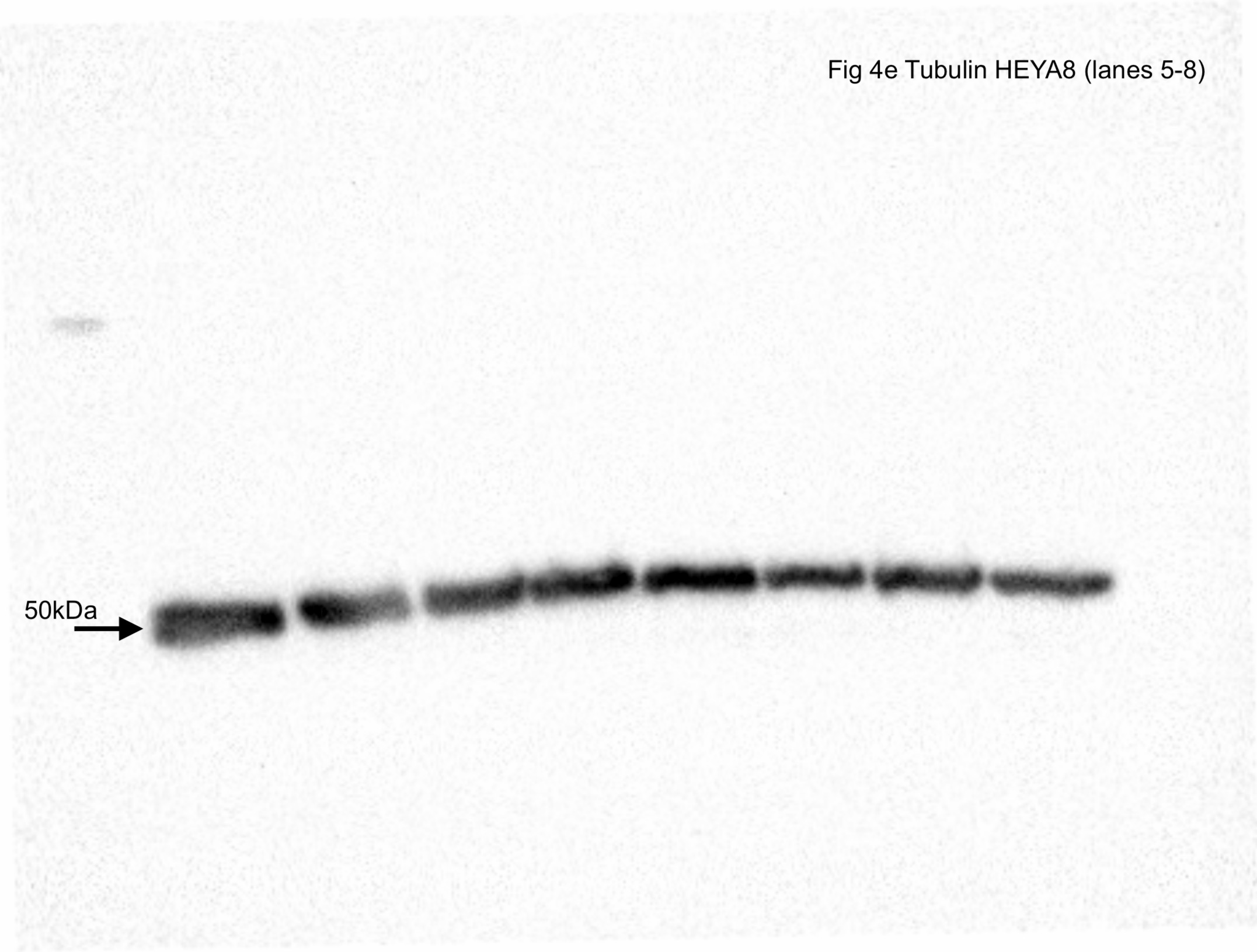


**Figure S44.** Whole blot image of Tubulin HEYA8 (lanes 5–8) from Figure 4E.


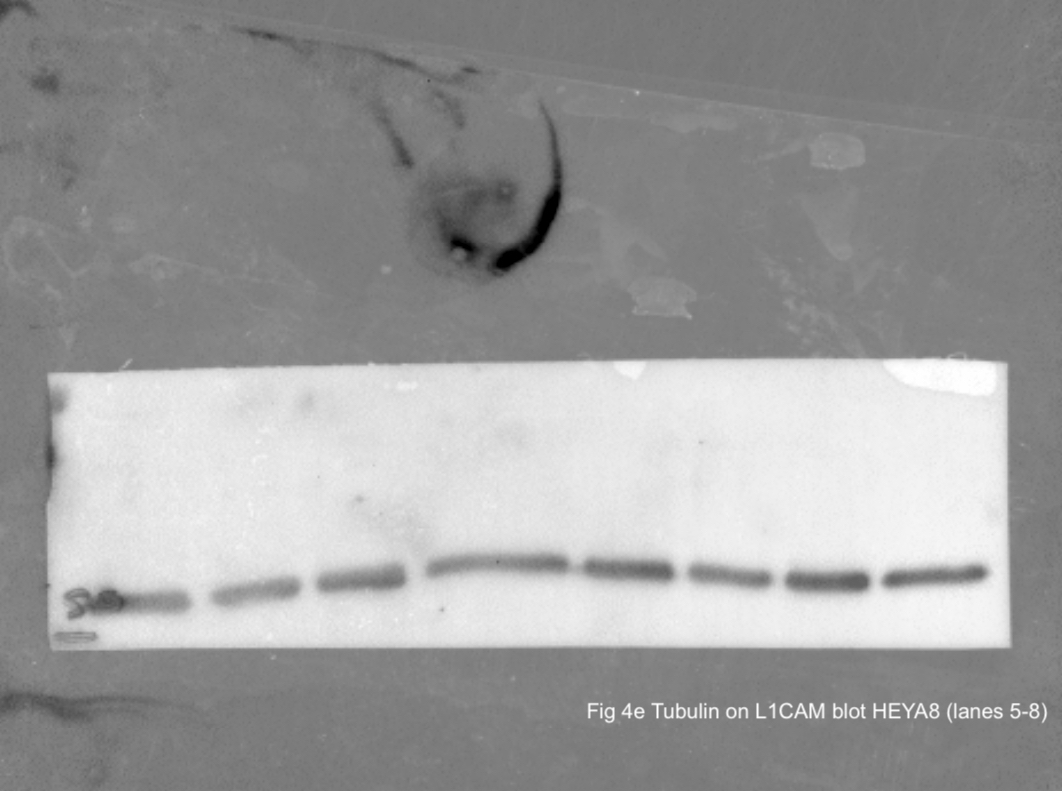


**Figure S45.** Whole blot image of Tubulin on L1CAM blot HEYA8 (lanes 5–8) from Figure 4E.

**Table S1** (separate Excel file “Table S1-S7.xlsx”): Primer sequences for qPCR.

**Table S2** (separate Excel file “Table S1-S7.xlsx”): Differentially expressed kinases (FDR < 0.05 from mapDIA) from MIB/MS analysis completed using OVCAR8 parental and OVCAR8 STK11-KO adherent cells.

**Table S3** (separate Excel file “Table S1-S7.xlsx”): Differentially expressed kinases (FDR < 0.05 from mapDIA) from MIB/MS analysis completed using OVCAR8 parental and OVCAR8 STK11-KO spheroids.

**Table S4** (separate Excel file “Table S1-S7.xlsx”): Differentially expressed genes from the Affymetrix Clariom S array using OVCAR8 parental and OVCAR8 NUAK1-KO spheroids.

**Table S5** (separate Excel file “Table S1-S7.xlsx”): Signatures from the GSEA Hallmark database that were enriched in the OVCAR8 parental spheroids compared to NUAK1-KO spheroids.

**Table S6** (separate Excel file “Table S1-S7.xlsx”): Signatures from the Curated Canonical database that were enriched in the OVCAR8 parental spheroids compared to NUAK1-KO spheroids.

**Table S7** (separate Excel file “Table S1-S7.xlsx”): Core enriched gene set from REACTOME_INTEGRIN_CELL_SURFACE_INTERACTION signature.
